# Active Potential of Bacterial Cellulose-Based Wound Dressing: Analysis of Its Potential for Dermal Lesion Treatment

**DOI:** 10.3390/pharmaceutics14061222

**Published:** 2022-06-08

**Authors:** Katharine Valéria Saraiva Hodel, Bruna Aparecida Souza Machado, Giulia da Costa Sacramento, Carine Assunção de Oliveira Maciel, Gessualdo Seixas Oliveira-Junior, Breno Noronha Matos, Guilherme Martins Gelfuso, Silmar Baptista Nunes, Josiane Dantas Viana Barbosa, Ana Leonor Pardo Campos Godoy

**Affiliations:** 1 SENAI Institute for Innovation in Advanced Health Systems (CIMATEC ISI SAS), SENAI/CIMATEC University Center, Salvador 41650-010, Brazil; katharine.hodel@fieb.org.br (K.V.S.H.); giulia.sacramento@fbter.org.br (G.d.C.S.); silmar@fieb.org.br (S.B.N.); josianedantas@fieb.org.br (J.D.V.B.); 2 Department of Clinical and Toxicological Analysis, Faculty of Pharmacy, Federal University of Bahia, Salvador 40170-115, Brazil; maciel.carines@gmail.com (C.A.d.O.M.); gessualdoj@ufba.br (G.S.O.-J.); leonor.godoy@ufba.br (A.L.P.C.G.); 3 Laboratory of Medicines, Food and Cosmetics (LTMAC), University of Brasília, Brasilia 70910-900, Brazil; brenomatos15@hotmail.com (B.N.M.); gmgelfuso@unb.br (G.M.G.)

**Keywords:** bacterial cellulose, wound dressing, dermal lesions, propolis, *p*-coumaric acid, biochanin A

## Abstract

The use of innate products for the fast and efficient promotion of healing process has been one of the biomedical sector’s main bets for lesion treatment modernization process. The aim of this study was to develop and characterize bacterial cellulose-based (BC) wound dressings incorporated with green and red propolis extract (2 to 4%) and the active compounds *p*-coumaric acid and biochanin A (8 to 16 mg). The characterization of the nine developed samples (one control and eight active wound dressings) evidenced that the mechanics, physics, morphological, and barrier properties depended not only on the type of active principle incorporated onto the cellulosic matrix, but also on its concentration. Of note were the results found for transparency (28.59–110.62T_600_ mm^−1^), thickness (0.023–0.046 mm), swelling index (48.93–405.55%), water vapor permeability rate (7.86–38.11 g m^2^ day^−1^), elongation (99.13–262.39%), and antioxidant capacity (21.23–86.76 μg mL^−1^). The wound dressing based on BC and red propolis was the only one that presented antimicrobial activity. The permeation and retention test revealed that the wound dressing containing propolis extract presented the most corneal stratum when compared with viable skin. Overall, the developed wound dressing showed potential to be used for treatment against different types of dermal lesions, according to its determined proprieties.

## 1. Introduction

Wounds have become one of the principal causes of death on a world level, resulting in great disorders in human health and economics developments, and representing a substantial financial burden for health systems [1]. With this context in mind, the expectations are that in 2021, the commerce of wound dressing will grow approximately 125% compared to in 2014, with exponential growth projections for the next years [2]. In Holland alone, it is believed that the total expenses directly related to the treatment of lesions costs around 2.4 billion euros (approximately $2.7 billion) per year [3]; meanwhile, in the United States, the estimated annual costs for chronic wounds treatment are over $33 billion [4]. This great financial movement in the lesion treatment market is mainly related to the high incidence of cases, as well as to the limitations of the widely available traditional treatments [5]. The absence of adequate treatment to promote the healing of these wounds can result in serious morbidities such as limb amputation [6]. Hence, the effective treatment of wounds is considered a major global health challenge, where the development of tools that can assist in the healing process can have a positive impact in decreasing financial and social stress for health governments authorities, care providers, patients, and their families [7]. 

Wound dressings play an essential role in healing as they provide a physical barrier between the wound and the external environment, thereby preventing further injury or infection by opportunistic microorganisms from occurring [8]. However, the use of traditional dressings, such as gauze and cotton, can have significant disadvantages when used to treat injured skin as they tend to adhere to wound sites, causing secondary injury during changes and leaving the microenvironment drier for tissue regeneration [9,10]. In this sense, efforts have been made to develop advanced materials (wound dressings) that are able to actively act during the healing process, reducing the treatment period and any possible health problems [11,12]. In Brazil, the use of advanced wound dressings in the treatment of skin lesions presented a cost seven times lower when compared to treatment with traditional dressings, as it decreased the time required for healing and, consequently, the number of changes of material needed [13].

The concept of modern or advanced wound dressings includes the use of materials that have specific properties–such as controlling the moisture of the lesion microenvironment–being able to remove excess exudate generated in the healing process, which allows thermal insulation and gas exchange, antimicrobial action, and adaptation to the injured surface [14,15]. Natural polymers, also called biopolymers, have been widely used in the regenerative therapeutic sector for the elaboration of wound dressings, since they comprise a group of high-performance materials of low environmental impact and wide availability. They also present a high biocompatibility with the extracellular matrix (ECM), resulting in a low cytotoxicity [16,17]. 

Cellulose is the world’s most abundant biopolymer, being a structural component present in the cell wall of plants and algae [18]. The cellulosic material obtained through acidophilic bacterial fermentation, known as bacterial cellulose (BC), has attracted the attention of the biopharmaceutical industry due to the absence of other biogenic products in its structure, such as hemicellulose, pectin, and lignin, which makes its purification process simpler and more cost-effective [19,20]. BC is formed by cellulose nanofibers (glucose monomers linked through β-(1, 4) glyosidic bonds) arranged randomly, which gives BC optimized properties compared to plant cellulose, such as high degree of polymerization, high mechanical strength in the wet state, high vapor permeability, high water absorption capacity, as well as in situ moldability [21,22]. The moldability of BC membranes is associated with the presence of electron-rich oxygen atoms in their large nanoporous surface area, which allows molecules with active functions to be incorporated into the cellulosic material in the intuit of enhancing or attributing new characteristics to the BC membrane, such as antimicrobial action [23,24]. 

In recent years, different authors have evidenced the efficiency related to the use of BC incorporated with natural components in accelerating the healing process when compared to treatment with traditional dressings, especially when analyzing the increase of antimicrobial activity, modulation of the immune system, and promotion of cell proliferation for tissue regeneration [25,26,27,28,29,30]. Among the natural components, propolis presents a great potential application for the treatment of injuries, since its 420 compounds–with a vast majority belonging to the group of phenolic compounds, flavonoids, and phenolic acids–are responsible for performing different biological activities [31] such as antimicrobial [32], antioxidant [33], and anti-inflammatory [34] action. Propolis originates from gummy, balsamic, and resinous substances, harvested by bees from buds, flowers, and exudates of plants, on which bees (such as the *Apis mellifera* species) deposit salivary secretions, wax, and pollen [35,36]. The composition of the plant where the bees take the material for the production of propolis determines its chemical composition, and this can vary according to the geographical location, which gives rise to different types of propolis [37,38]. 

Green and red propolis are the main types studied and commercialized in Brazil due to their specific chemical markers (also called biomarkers), which give them superior biological activities to the other 12 types of propolis cataloged in Brazil [39,40]. In the case of green propolis, the prenylated compounds and cinnamic acid derivatives–such as *p*-coumaric acid (4-hydroxycinnamic acid) [41,42]–stand out, while in red propolis it is isoflavones such as biochanin A (5,7-dihydroxy-4′-methoxyisoflavone) [43,44] that are of note. Thus, it is important to emphasize that the biological properties of propolis should not be considered only as a synergistic effect of its components, since there is a need for isolation and identification of the bioactive constituents responsible for the biological effects widely associated with the use of propolis [45,46,47]. 

Accordingly, the aim of this study was to develop bacterial cellulose-based wound dressings incorporated with green and red propolis extracts and the active compounds *p*-coumaric acid and biochanin A, and to characterize the materials obtained with respect to morphological, optical, physicochemical, and biological properties, aiming at their potential application as a wound dressing for the treatment of dermal lesions.

## 2. Materials and Methods

**Figure 1:**
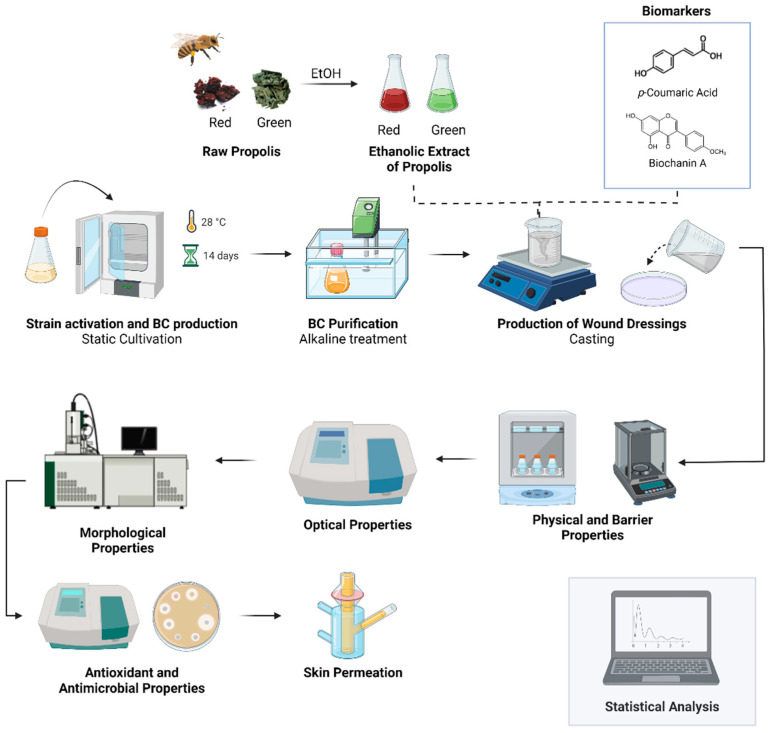
Overview of the production steps and characterization of wound dressing based on BC and the ethanolic extract of green and red propolis, *p*-coumaric acid, and biochanin A. Image created with Biorender.com (accessed on 30 March 2022).

The main steps of the methodology used to produce and characterize the BC-based wound dressing and the active substances (ethanolic extract of green and red propolis, *p*-coumaric acid, and biochanin A) are presented in Figure 1.

### 2.1. Obtaining and Processing the Raw Propolis

The raw propolis samples used in this project were donated by local beekeepers; the red propolis came from the city of Belomonte (−15.8596, −38.8903) in the state of Bahia, and the green propolis came from the city of Queluzito (−20.7354, −43.8767), state of Minas Gerais. About 1000 g of the samples were ground in a mill (Cadence; Balderário das Piçarras, Balneário Piçarras, Santa Catarina, Brazil) and sieved (60 mesh) to obtain a particle size of approximately 0.180 mm, allowing an increase in the surface area and a homogenization of the starting material for the extraction processes. In order to avoid degradation of the material, propolis samples were fractionated into small amounts (250 g) and were kept in the freezer at −10 °C, in flasks protected with laminated paper in inert (N2) atmospheric conditions [48].

### 2.2. Production of the Ethanolic Extracts of Green and Red Propolis

To obtain the ethanolic extracts (EtOH), propolis samples (2 g) were ground and homogenized with 25 mL of ethanol (80:20 v v^−1^) [49,50,51]. Subsequently, the samples were heated in a water bath (521-7TD; Ethik Technology; Vargem Grande Paulista, São Paulo, Brazil) under mechanical stirring for 30 min at 50 °C, followed by centrifugation at 8800 rpm for 10 min at 5 °C [52]. The supernatant resulting from centrifugation was filtered on quantitative paper (80 g cm^−3^) and then transferred to glass vials, where its contents were concentrated at 40 °C (miVac-DUC-22060-N00; Genevac; Palo Alto, Santa Clara, CA, USA) until constant weight. The obtained extract was stored at −18 °C and its container was wrapped with laminated paper to avoid degradation until the moment of incorporation into formulations to obtain the active wound dressing.

### 2.3. Production and Purification of Bacterial Cellulose Membranes

The *Glucanoacetobacter hansenii* strain (ATCC 23769), obtained from the Tropical Culture Collection (CCT)—André Tosello Foundation, was used in the fermentation process under static conditions to obtain BC membranes. The culture medium for the preparation, activation of the inoculum, and formation of the biopolymer had the following composition: 50 g L^−1^ glucose, 5 g L^−1^ yeast extract, 3 g L^−1^ peptone, and 2 g L^−1^ potassium phosphate (KH_2_PO_4_) [53]. Approximately 20 µg of *G. hansenii* biomass was inoculated into the culture medium after being sterilized in an autoclave (121 °C, 1 atmospheric pressure, 15 min), and was then incubated in a bacteriological oven (0316M2; Quimis; Diadema, São Paulo, Brazil) at a temperature of 28 ± 2 °C for 14 days. After the incubation period, the BC membrane produced on the air/medium surface was removed and purified through an alkaline treatment with potassium carbonate (K_2_CO_3_) (0.3 mol-L^−1^) and thermal treatment (80 °C), as described in previous studies [53,54]. After the purification process, the membranes were kept in deionized water at 4 °C until their next use.

### 2.4. Preparation of the Wound Dressings

The purified BC membranes were ground in a multiprocessor and homogenized until a gel was obtained, to be later used in the production of the wound dressings. The formulations were prepared according to the experimental design of Table 1, with a fixed amount of BC gel and variations in the amount of propolis extracts and isolated standards, *p*-coumaric acid (Sigma-Aldrich, St. Louis, MO, USA), and biochanin A (Sigma-Aldrich, St. Louis, MO, USA), totaling nine formulations produced using the casting technique [55]. The determination of the maximum concentrations of each component was performed from preliminary tests, where the formation of flexible, easy-to-handle, and homogeneous structures (films) was evaluated. In addition, the values were based on works published in the literature, such as the study by Picchi [56], which demonstrated that BC membranes with at least 2.0 g of dry mass of propolis presented a satisfactory antimicrobial activity for application as a wound dressing. The values of the standards were established according to the study of Machado et al. [57] for the green propolis sample, where the value found for *p*-coumaric acid was approximately 8.0 mg, the same value found for biochanin A in the red propolis samples.

For the preparation of the filmogenic solution, 50 g of the BC gel and active solutions at the concentrations shown in Table 1 were kept under stirring at 60 rotation per minute (rpm) (C-MAG HS7; IKA, Staufen, Germany) for 10 min, or until a homogeneous solution was obtained. The filmogenic solution was placed in polystyrene Petri dishes and dehydrated in a forced air circulation oven at a temperature of 45 ± 2 °C (Q314M222; Chemis; Diadema, São Paulo, Brazil) for 24 h or until constant weight. After dehydration, the wound dressings were kept in a vacuum dissector at a temperature of 20 ± 2 °C in a 60% relative humidity atmosphere until their properties were analyzed.

### 2.5. Characterization of the Wound Dressings

#### 2.5.1. Scanning Electron Microscopy (SEM) of the Wound Dressings

To perform SEM, the samples were manually fixed and metallized with gold as reported by Machado et al. [48] and Nunes et al. [54]. After processing, the samples were analyzed with a scanning electron microscope (SEM, BX-51; Olympus; Tokyo, Japan) at different magnifications (voltage 12 kV, working distance 12 mm, spot size 44, HV vacuum mode).

#### 2.5.2. Opacity and Transparency of the Wound Dressings

The opacity and transparency of the wound dressings were determined by spectrophotometric techniques (700 PLUS; FEMTO; São Paulo, Brazil), where the specimens were cut in a rectangular shape (1.5 × 4 cm) and the material was embedded in the internal wall of the quartz cells to be analyzed. The opacity was defined from the absorbance value at 500 nm, divided by the thickness (mm) (Abs_500_ nm mm^−1^) [58]. Transparency, on the other hand, was defined by the ratio between the transmittance at 600 nm divided by the thickness (mm) [59]. Opacity and transparency were measured in triplicate.

#### 2.5.3. Grammage and Thickness of the Wound Dressings

The grammage of the wound dressings was determined from the ratio of the mass of the samples divided by the total area (2 cm^2^) [54,58]. The thickness of the wound dressings was defined from 10 random points on each sample using a flat-tip digital micrometer (Ip40; Digimess; São Paulo, Brazil) at a setting of 0.001 mm.

#### 2.5.4. Water Activity (aw) and Water Solubility of the Wound Dressings

The aw of the wound dressings was determined using a CM-2 electrolytic measuring cell in the Decagon (Lab Master aw; Novasina; Lachen, Switzerland) at room temperature (25 ± 2 °C) with 4 cm^2^ samples, as performed by Leal et al. [60]. For water solubility analysis, samples of each wound dressing with 2 cm in diameter were weighed (initial mass or m0) and placed in Erlenmeyer flasks for immersion in 50 mL of distilled water. The flasks containing the samples were shaken at 130 rpm at a temperature of 25 ± 2 °C for 24 h in an incubator with an orbital shaker (MA420; Marconi; Piracicaba, São Paulo, Brazil). After this step, the insoluble part of the samples was dried (105 ± 2 °C 24 h) in an oven with forced air circulation (Q314M222; Quimis; Diadema, São Paulo, Brazil) and subsequently weighed again (final mass or m_f_) [61]. Water solubility values were determined from the m_o_ and m_f_ values in percentage (%), according to Equation (1). Water activity, grammage, and water solubility were evaluated in triplicate.

Water solubility (%) = ((m_o_ − m_f_))/m_o_ × 100 (1)

#### 2.5.5. Index of Swelling and Moisture Content of Wound Dressings

The swelling index and moisture content of the wound dressings were determined using the gravimetric method as presented by Du et al. [62], with modifications. Initially, the wound dressings were cut into samples with an area of 16 cm^2^ and the values of their respective masses were set (m_dry_). Each sample was immersed in a beaker containing 30 mL of deionized water and kept at room temperature (25 ± 2 °C). After being immersed for 24 h, the intumesced samples were removed from the water and, using a filter paper, the excess water on the surface was removed and the mass of the samples was measured again (m_moist_). The swelling index (%) and the moisture content (%) of the wound dressings were defined by Equations (2) and (3), respectively. The analyses were performed in triplicate.

Index of intumescence (%) = ((m_moist_ − m_dry_))/m_moist_ × 100 (2)

Moisture content index (%) = ((m_moist_ − m_dry_))/m_moist_ × 100 (3)

#### 2.5.6. Thickness, Water Vapor Transmission Rate (WVTR), and Water Vapor Permeability (WVP) of the Wound Dressings

The WVTR and WVP of the wound dressings were determined by gravimetry according to the standard method presented in ASTM E96-00, with adaptations (ASTM, 2000). The wound dressings (F1–F9) were cut into circular samples (~60 mm in diameter) and placed and sealed in their respective permeation cells, which contained distilled water (30 mL). The cells containing the samples were placed in a desiccator that contained silica gel (relative humidity 0% and 25 °C) to ensure a water gradient in the system. Over seven days, the weight of the cells and samples was monitored at 24 h intervals in order to follow the variation of mass over time. The WVTR was determined from Equation (4), while the WVP was obtained through Equation (5), and both analyses were performed in triplicate.

WVTR (g/m^2^ day) = ∆m/∆t × A (4)

WVP (10^−8^ g-mm/m^2^-day-Pa) = TTVA × E/ΔP (5)

where: WVTR is the water vapor transmission rate (g/m^2^ day) of the wound dressing (samples F1 to F9); PVA is the water vapor permeability (10^−8^ g·mm/m^2^·day·Pa) of the wound dressing (samples F1 to F9); ∆m is the is the permeation cell weight change (g); E is the average thickness of the wound dressing (mm); ∆t is the elapsed time (day); A is the permeation area of the samples (mm); ΔP is the vapor pressure difference of the environment containing silica gel (kPa, at 25 °C) and pure water (3.167 kPa, at 25 °C) g t^−1^.

#### 2.5.7. Tensile Mechanical Properties of the Wound Dressings

Tensile tests were performed in order to determine the maximum tensile strength (MPa) and elongation (%) of the wound dressings. The tests were performed according to ASTM D-882 (ASTM, 2018), with adaptations, using a texturometer (CT310k; Brookfield; Phoenix, AZ, USA). Seven specimens of each sample (100 mm × 25 mm) were analyzed after being conditioned in dissectors with saturated sodium chloride solution (58% relative humidity, 25 °C) for 48 h. After this period, the specimens were placed between the test tips of the equipment (TA3/100 and TA/TPB) at an initial distance of 50 mm, and tractioned at a speed of 50 mm min^−1^.

#### 2.5.8. Evaluation of Total Flavonoid Content, Total Phenolic Compounds, and Antioxidant Action of the Wound Dressings

About 250 mg of each wound dressing was placed in plastic centrifuge tubes capped with 25 mL of 80% ethanol (v v^−1^). The tubes were centrifuged at 1000 rpm for 10 min at 25 °C and the supernatant liquid was transferred to a set of glass tubes for further storage at 4 °C. Before 24 h has passed since processing, the supernatant liquid was used to determine total flavonoid and phenolic content, as well as antioxidant activity, by sequestering the 2,2-diphenyl-1-picrylhydrazyl radical (DPPH).

The total flavonoid content (TFC) of the wound dressings was determined according to the method proposed by Meda et al. [63], with adaptations. Initially, 2.0 mL of each sample (0.5 mg mL^−1^) was transferred to the test tube and added to 2.0 mL of 2% (m v^−1^) methanolic solution of aluminum chloride (AlCl_3_). After homogenization and maintenance under the shelter of light for 30 min (25 °C), the solution (sample: aluminum chloride) were read in triplicate at 415 nm absorbance (700 PLUS; FEMTO; São Paulo, Brazil). The same procedure was performed using known solutions of quercetin (from 5 to 105 µg mL^−1^) to prepare a standard curve (y = 0.0311x + 0.0259, R^2^ = 0.9987). The amount of total flavonoids was expressed as quercetin equivalents per gram of sample (mgQE g^−1^).

The total phenolic content (TPC) of the nine wound dressings was determined using methodology by Singleton and Rossi [64] and Singleton et al. [65], and was based on the reaction with Folin-Ciocalteu reagent, which is associated with the appearance of a blue color due to phenol oxidation in a basic medium [66]. Following the reaction with Folin-Ciocalteu aqueous solution (at 10% concentration) and sodium carbonate (at 7.5% concentration), the supernatant liquid of the wound dressings was heated at 50 °C for 5 min. After this procedure, the mixture was read in the spectrophotometer ((700 PLUS; FEMTO; São Paulo, Brazil) at the absorbance of 765 nm with a quartz cuvette. The same procedure was performed using known solutions of gallic acid (from 12 to 200 µg mL^−1^) to prepare a standard curve (y = 0.0104x + 0.0688, R^2^ = 0.9976). The amount of TPC was expressed as gallic acid equivalents per gram of sample (mgGAE g^−1^), and it was performed in triplicate.

The antioxidant capacity of the wound dressings was determined from DPPH (2,2-diphenyl-1-picrylhydrazyl) radical sequestration analysis, according to the methodologies proposed by Brand-Williams et al. [67] and Molyneux [68], with modifications. For this, six dilutions of each sample were prepared at concentrations from 10 to 85 μg mL^−1^ (in triplicate). Then, a 1 mL aliquot of each dilution of the samples was transferred to test tubes containing 3.0 mL of ethanolic solution (99%, v v^−1^) of the DPPH radical (0.004%, m v^−1^) (Sigma-Aldrich, St. Louis, MO, USA). After incubation for 30 min in the dark (25 °C), the samples were read at 415 nm absorbance (700 PLUS; FEMTO; São Paulo, Brazil), where the blank considered was the ethanol solution. Equation (6) was used to calculate the ability of the samples to sequester the free radical expressed as a percentage of the inhibition of radical oxidation. The IC_50_ value (concentration required of the extract to sequester 50% of the DPPH radical) was calculated using the linear equation based on the concentrations of the samples in their respective percentages of sequestering the DPPH radical (µg mL^−1^) (Equation (6)).

% DPPH radical sequestration = 100 − [(final absorbance of the sample × 100)/absorbance of the blank](6)

#### 2.5.9. Antimicrobial Activity of the Wound Dressings

The *Staphylococcus aureus* (ATCC 6538) and *Escherichia coli* (ATCC 8739) antimicrobial activity of the wound dressings was determined from the disc diffusion method (Clinical and Laboratory Standards Institute (CLSI)) [69] with modifications. The bacterial suspensions were inoculated in a sterile saline solution (0.9%) until they reached a turbidity equivalent to 0.5 of the McFarland scale (approximately 108 CFU mL^−1^). A sterile swab was used to seed the suspension onto the surface of Mueller-Hinton agar in a Petri dish (90 × 15 mm). The wound dressings were cut into discs (diameter 5 mm) and exposed to ultraviolet light for 20 min (per side) using a laminar flow cabinet to sterilize the surface. In each Petri dish, three (3) discs of each wound dressing were tested, the sample F1 was used as a negative control, and the antibiotic disc (Penicillin G for *S. aureus* and Cephalosporin for *E. coli*) was used as a positive control. The plates were incubated at 35 °C aerobically for 18 h. After this period, the diameters of the inhibition zones were measured using a measuring tool.

### 2.6. In Vitro Skin Permeation Analysis of Wound Dressings

The in vitro skin permeation analysis was performed using Franz diffusion cells (diffusion area = 1.7 cm^2^), which were mounted with pig ear skin separating the donor compartment from the receptor compartment. The recipient compartment was filled with 0.1 M phosphate buffer solution, pH 7.4. For 10 min, the skin was left in contact with the receptor solution with the intention of hydrating it before contact with the wound dressings. In the donor compartment the active wound dressings (samples F2–F9) were added. The receiver solution was kept at a temperature of ±33 °C and stirred at 500 rpm for 2 h. Samples of the receptor fluid were collected at four time points during the 2 h of passive permeation, and subsequently analyzed by High Performance Liquid Chromatography (HPLC). The analytical method for the quantification of *p*-coumaric acid and biochanin A, as well as its validation and standardization, are presented in Appendix A.

#### Determination of the Amount of *p*-Coumaric Acid and Biochanin A Retained in the Stratum Corneum and Viable Skin

After the skin permeation experiment, the amount of biomarkers (*p*-coumaric acid and biochanin A) that remained retained in the stratum corneum of the skin was determined and differentiated from the content retained in the viable skin. For this, the tape stripping technique was used, where the pig skin was removed from the diffusion cell, stretched, and stuck on a Styrofoam support. The stratum corneum of the drug transport area was completely removed with the help of 15 tape strips, and the viable skin was minced. Both layers were transferred to amber glass vials, from which the markers were extracted by the addition of 5 mL of methanol. The flasks were capped and left to stand for 24 h at room temperature (25 °C). After this period, their content was filtered on hydrophobic filters with porosity of 0.45 µm and analyzed by HPLC for quantification of *p*-coumaric acid (for samples F2, F3, F6, and F7) and biochanin A (for samples F4, F5, F8, and F9), the biomarkers under study.

### 2.7. Statistical Analysis

All data from the characterization assays were presented as mean values ± standard deviation, since at least three independent analyses were performed. The results obtained related to the characterization of wound dressings were analyzed for 95% (*p* < 0.05) significance, and results that showed significant differences between treatments were differentiated by Tukey’s post-test. Analysis of statistical significance was determined by ANOVA and Tukey-test. To analyze the influence of the insertion of the active substances (propolis extracts or the isolates), the principal component analysis (PCA) was performed, using the means of the characterization analyses (aw, weight, water solubility, swelling index, moisture content index, WVTR, WVP, opacity, transparency, thickness, elongation, tensile strength, TFC, TPC, and DPPH radical scavenging capacity). However, since the variables evaluated had different units of measurement, the data for the aforementioned characterization tests were normalized in the range of 0 to 1, and after normalization, PCA was performed. Considering the in vitro retention analysis, the comparison between the concentration of biomarkers (*p*-coumaric acid and biochanin A) in the stratum corneum and in the viable skin through passive permeation of the wound dressings was carried out using the Bonferroni test. All statistical analyses were performed in GraphPad Prism software (version 9.2; San Diego, CA, USA).

## 3. Results

### 3.1. Production Kinetics of the BC, Visual Appearance, and Morphological Properties of Active or Pure BC Wound Dressings

**Figure 2:**
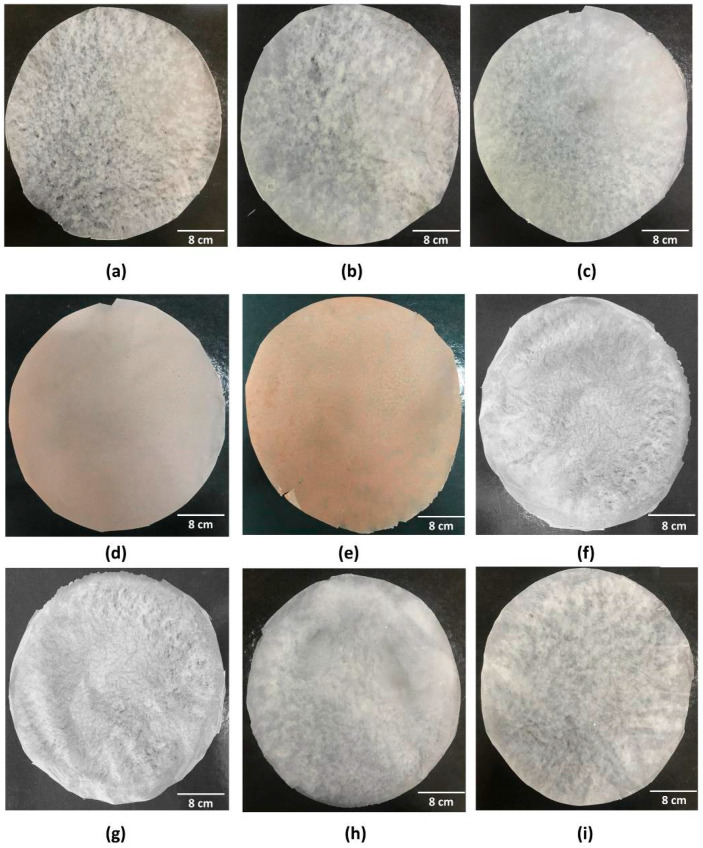
Visual appearance of the nine wound dressings based on pure BC or BC incorporated with active molecules: (**a**) F1; (**b**) F2; (**c**) F3; (**d**) F4; (**e**) F5; (**f**) F6; (**g**) F7; (**h**) F8; and (**i**) F9.

Nine wound dressings (pure BC or with active substances) were produced from static culture of BC with modified HS medium. Figure S1a presents the BC production kinetics, based on the OD_(600 nm)_ and total soluble solids concentration (°Brix) during 14 days of fermentation, where it can be seen that the OD_(600 nm)_ increased while the °Brix decreased over time. As can be seen in Figure S1b, the BC membranes obtained after 14 days of fermentation showed about 15.85 g ± 0.134 after purification. From the BC membranes produced, nine (9) wound dressings were obtained by the casting technique after total drying of the samples, which may vary in the presence and/or concentration of active substances (ethanolic extract of propolis or isolated molecules) (Table 1). Thus, the wound dressings presented distinct morphologies, which can be associated with their different components (Figure 2 and Figures S2–S10). The wound dressings, regardless of their composition, were removed from the Petri dish surface without breaking their structure, which facilitated their handling in obtaining samples for characterization tests. As reported by Barud et al. [70], from the incorporation of ethanolic extract in BC membranes, the wound dressings started to present amber colors in a dose-dependent manner. However, it is important to emphasize that the incorporation of propolis isolates (*p*-coumaric acid and biochanin A) promoted the obtaining of wound dressings with a similar appearance to pure BC (sample F1). 

**Figure 3:**
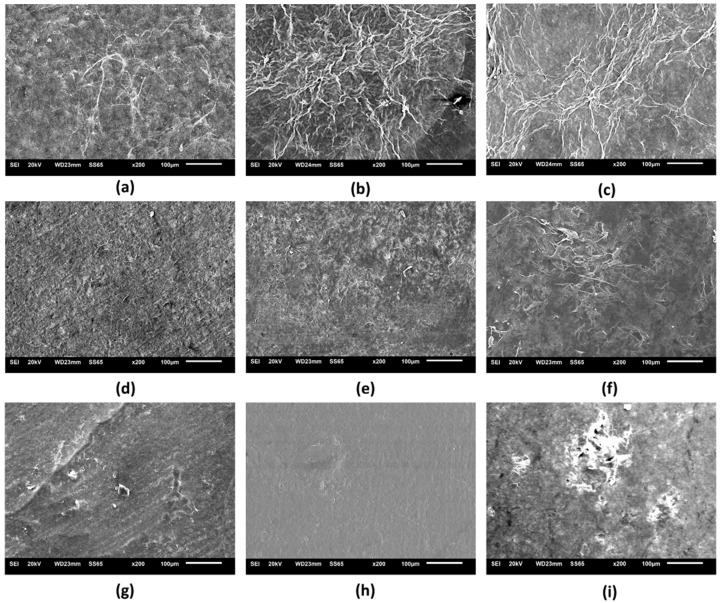
Scanning electron microscopy micrographs (200×) of the surface of the nine wound dressings based on pure BC or BC incorporated with active molecules: (**a**) F1; (**b**) F2; (**c**) F3; (**d**) F4; (**e**) F5; (**f**) F6; (**g**) F7; (**h**) F8; and (**i**) F9.

Figure 3 and Figures S11–S19 shows the micrographs of the nine (9) wound dressings developed, considering the pure BC (sample F1) or through the incorporation of different active substances in its matrix. Micrographs of samples F1, F2, and F3 showed that these materials have an irregular distribution of cellulose fibers, and micrographs of samples F2 and F3 evidenced the possible appearance of porous regions. Bodini et al. [71] reported that the presence of ethanolic extract of propolis in gelatin films resulted in an irregular surface, with porous regions that were possibly formed due to the distribution of the ethanolic extract in the matrix. However, the incorporation of the ethanolic extract of red propolis (samples F4 and F5) into BC led to the formation of a homogeneous matrix. Importantly, the *p*-coumaric acid content present in sample F7 (Figure 3g) was able to interact with the cellulose fibers modifying the polymeric base when compared to sample F1 formed by BC alone (Figure 3a). The study by Yong et al. [72] demonstrated through the SEM technique that *p*-coumaric acid was able to interact with chitosan polymers through intermolecular hydrogen bridges, resulting in a more compact film. A similar effect may have occurred with sample F7, where the higher concentration of *p*-coumaric acid may have increased the amount of hydrogen bridges, allowing a greater opportunity for interaction between the active ingredient and the polymer compared to sample F6. Figure 3h (sample F8) and 3i (sample F9) showed that the concentration of biochanin A was an important factor for defining the microstructure of the material, where lower content of this active ingredient (8 mg) resulted in greater cohesion with the BC, while the highest concentration (16 mg) possibly resulted in the formation of agglomerates.

### 3.2. Characterization of Active and Pure BC Wound Dressings: Optical Properties

**Figure 4:**
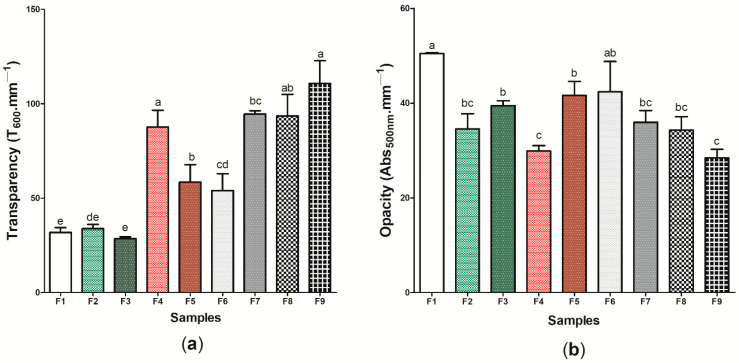
Optical properties of wound dressings F1 to F9: (**a**) transparency (T_600_ mm^−1^) (**b**) (Abs_500_ mm^−1^). Bars followed by the same letters ^(a,b,c,d,e)^ were not significantly different considering <0.05 as *p* value according to Tukey’s test with 95% confidence level.

Figure 4 presents the optical properties of the nine (9) wound dressings produced, which was evaluated from the analysis of transparency (Figure 4a) and opacity (Figure 4b) (Table S1). Considering the analysis of transparency, wound dressing F1 presented one of the lowest values obtained for this analysis (31.81 ± 2.57 T_600_ mm^−1^), however, wound dressings F4 and F9 presented the highest transparency, with 87.72 ± 8.771 and 110.93 ± 12.07 T_600_ mm^−1^, respectively. Andriotis et al. [73] demonstrated that lower concentrations of propolis extract in the pectin polymeric matrix promoted the obtaining of more transparent bioactive adhesives, as also observed in this study. Furthermore, the results found in Andriotis et al.’s study are similar to the pattern reported by Contardi et al. [74], where increasing the concentration of *p*-coumaric acid in polyvinylpyrrolidone (PVP)-based films led to increased transparency. If it comes to the opacity of wound dressings, the values found ranged from 50.48 ± 0.152 (sample F1) to 28.43 ± 1.804 (sample F9) Abs_500_ nm-mm^−1^, with a significant difference (*p* < 0.05). 

Eskandarinia et al. [75] showed that there was an increase in the opacity of wound dressings based on corn starch, hyaluronic acid, and propolis as the concentration of propolis extract also increased, with values between 1 and 5 Abs_500_ mm^−1^, which differs from the results found in this study for this property. The high opacity in the sample F1, containing only BC, may be related to the properties of microscaled cellulosic fibers, which have low transparency and reflect more light [76]. Thus, the presence of other components in the BC matrix may have altered the material’s ability to reflect light. Moreover, it is important to note that the transparency of a dressing allows the healing process to occur without the need for successive changes of the material, thus decreasing the likelihood of secondary lesions and, consequently, point of infections of opportunistic microorganisms; this aids the treatment of superficial dermal lesions and generates a low production of exudates [77,78]. In this case, samples F4, F7, F8, and F9 presented the highest potential for application considering this perspective.

### 3.3. Characterization of Active and Pure BC Wound Dressings: Physical and Barrier Properties

**Figure 5:**
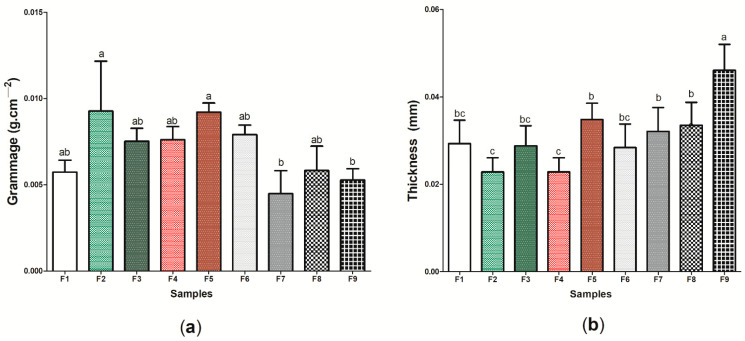
Physical and barrier properties of wound dressings F1 to F9: (**a**) grammage (g cm^−2^) and (**b**) thickness (mm). Bars followed by the same letters ^(a,b,c)^ were not significantly different considering <0.05 as *p* value according to Tukey’s test with 95% confidence.

Figure 5a shows the results regarding the grammage of the nine (9) wound dressings produced, where the values found ranged between 0.004 ± 0.001 g cm^−2^ (wound dressing F7) and 0.009 ± 0.002 g cm^−2^ (wound dressing F9) (Table S1). It is important to note that there was no significant difference (*p* < 0.05) between the wound dressings composed of pure BC and the other eight (8) wound dressings formed from the incorporation of active components to BC, which demonstrates that the presence of ethanolic extract of propolis (green and red) or its biomarkers (*p*-coumaric acid and biochanin A) was not able to significantly modify the distribution of mass in a respective area. This difference may be associated with the type of molecule that was incorporated into the BC matrix, where biopolymer incorporation may result in an interaction. Considering the thickness analysis (Figure 5b), wound dressing F9 presented the greatest thickness among the nine (9) samples produced (0.0461 ± 0.005 mm), while the samples containing 2 g of green or red propolis extract had the lowest thickness, 0.0269 ± 0.004 and 0.0228 ± 0.004 mm, respectively, with a significant difference (*p* < 0.05) (Table S1). It is important to note that, in general, comparing sample F1 with active wound dressings (samples F2–F9), there was only a statistical difference for sample F9. 

This behavior was similar to that reported by Marques de Farias et al. [79], where the presence of propolis extract, regardless of its concentration, did not affect the thickness of cassava starch films (*p* > 0.05), and the results found ranged from 0.05 ± 0.00 to 0.09 ± 0.1 mm. The thickness observed in sample F9 may be related to the interaction between biochanin A and BC, where a greater amount of the active ingredient may have acted as an additive, promoting the intumescence of the material and, consequently, the increase in thickness. Importantly, thickness is a property that can correlate with other important characteristics for an “ideal” wound dressing, since through this property it is possible to control the permeability for fluids and gases, as well as the mechanical and optical properties of the material [80].

**Figure 6:**
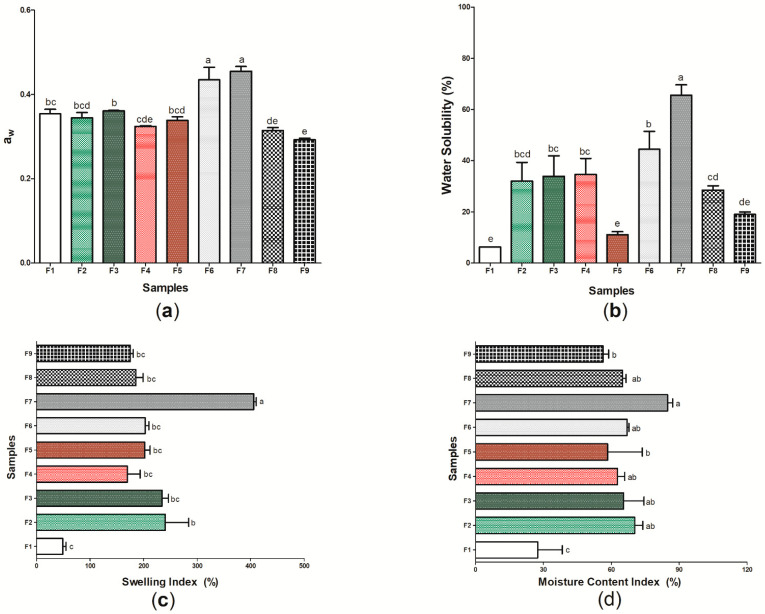
Physical and barrier properties of wound dressings F1 to F9: (**a**) aw; (**b**) water solubility (%); (**c**) swelling index (%); and (**d**) moisture content index (%). Bars followed by the same letters ^(a,b,c,d,e)^ were not significantly different considering <0.05 as *p* value, according to Tukey’s test with 95% confidence.

Figure 6 shows the results for aw (Figure 6a), water solubility (%) (Figure 6b), swelling index (Figure 6c), and moisture content index (Figure 6d) of the nine (9) wound dressings produced (Table S1). The aw between the samples ranged from 0.293 ± 0.003 (sample F9) to 0.455 ± 0.011 (sample F7). When compared to pure BC (sample F1), wound dressings F6 and F7, which have *p*-coumaric acid as active substance, presented higher values for aw, with a significant difference (*p* < 0.05). However, the addition of biochanin A resulted in decreased aw when compared to sample F1 (*p* < 0.05). Water activity is a property that determines the amount of free water in a sample, where values >0.85 indicate a greater propensity to bacterial growth due to high humidity [81]. Thus, the values found in this study (0.293 to 0.0455) show a lower susceptibility to bacterial proliferation, which is desirable for the wound healing process [82]. Furthermore, different studies have demonstrated the importance of values for aw <0.600, as the ones obtained in this study, since they promote a balance in the moisture content of the material and are not able to decrease WVP, thus allowing the control of moisture in the microenvironment of the lesion [83,84].

The sample F1 presented the lowest water solubility among the nine samples evaluated, with 6.25 ± 1.087%. In general, the incorporation of propolis extracts or isolated patterns in the cellulosic matrix increased the water solubility of the wound dressings when compared to pure BC (Figure 6b). Water solubility is associated with the hydrophilicity of the material [85], and thus this characteristic may have been modified through the structural changes found from samples F2 to F9 when compared to sample F1. The highest solubilities found in this study were 65.59 ± 4.073% and 44.49 ± 6.969% found in samples F7 and F6, respectively. These results may be associated with the presence of glycosidic bonds with the hydroxyl group of *p*-coumaric acid, which increases its interaction with water and may facilitate solubilization [86]. Considering samples F8 and F9, different studies show that biochanin A has a low aqueous solubility, which may have reflected in a lower solubility when compared to *p*-coumaric acid [87,88]. The ethanolic extracts of green and red propolis, despite having hydrophilic compounds in their composition, also have a hydrophobic character, mainly represented by the presence of wax in their composition [89]. The more soluble nature of a dressing may be related to its application in cases where the treatment of the lesion must be performed on sensitive and damaged skin, since this characteristic may be important for the removal of the material to be performed without abrasion to the skin, which demonstrates the potential application of samples F6 to F7 [90].

In addition to the analysis on aw and water solubility, the determination of the swelling index (Figure 6c) and the moisture content index (Figure 6d) of the F1–F9 samples allowed a better understanding of the hydrophilic or hydrophobic character of the wound dressings. Sample F1 was the one that presented the lowest swelling index (48.93 ± 6.02%), as well as the lowest index of moisture content (27.44 ± 10.819%), while sample F7 (based on BC and 16 mg of *p*-coumaric acid) was the one that presented the highest values for these two characterizations, 86.00 ± 2.372% for moisture content and 405.55 ± 4.81% for the swelling index. The interaction of *p*-coumaric acid with water, as shown in the solubility analysis (Figure 6b), may have facilitated the temporary insertion of the water molecule into the polymer chain, thus allowing the intumescence of the material until an equilibrium relationship occurs [58]. In general, the swelling index can indicate whether a dressing has the ability to absorb exudates, preventing the accumulation of electrolytes, nutrients, immune mediators, as well as waste products that may be harmful to the healing process [91,92]. Values for the intumescence index between 100 and 900% are considered ideal for an “ideal” wound dressing [93], which suggests that active wound dressings (samples F2–F9) have potential application for burn-type injuries, which have as one of the main consequences of their healing physiology the high release of exudates [94].

**Figure 7:**
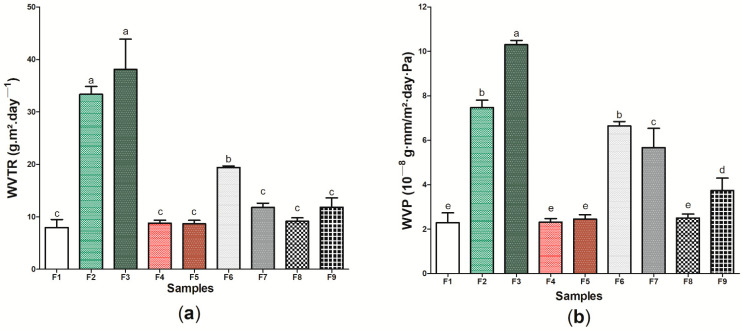
Physical and barrier properties of wound dressings F1 to F9: (**a**) WVTR (g m^2^ day^−1^) and (**b**) WVP (10^−8^ g-mm/m^2^-day-Pa). Bars followed by the same letters ^(a,b,c,d,e)^ were not significantly different considering <0.05 as *p* value according to Tukey test with 95% confidence level.

Furthermore, an “ideal” wound dressing should present a WVTR or WVP capable of promoting an adequate microenvironment for the adequate recovery of the injury, where dressings with a high WVTR or WVP may cause skin dryness, increasing the probability of scar formation, and lower values may cause the accumulation of exudates [95,96]. With this perspective, Figure 7 presents the WVTR (Figure 7a) and WVP (Figure 7b) results of the wound dressings formed by bacterial cellulose (sample F1) and the eight (8) samples produced from the incorporation of active components to its matrix (Table S1). Samples F2 and F3 formed by BC and ethanolic extract of green propolis were those that presented the highest WVTR, with 33.37 ± 1.116 and 38.12 ± 4.265 g m^2^ day^−1^, respectively, while sample F1 presented the lowest value for this property, 7.97 ± 1.184 g m^2^ day^−1^. The same behavior was observed for WVP analysis, where samples F2 and F3 presented the highest results, 10.30 ± 0.195 and 7.48 ± 0.329 10−8 g-mm/m^2^-day-Pa, respectively, while the sample formed by pure BC presented the lowest WVP, 2.29 ± 0.445 10^−8^ g-mm/m^2^-day-Pa.

WVTR and WVP can be influenced, among other factors, by the internal structure of the material [97,98]. The high phenolic compound content of the red propolis extract [48,99] may have interacted with the hydroxyl groups present on the BC surface, causing the permeability of the wound dressings not to be significantly modified (*p* > 0.05) when compared to the sample F1 [100]. This interaction causes covalent or hydrogen bonds to become established, decreasing the interaction of wound dressings with water. The decrease in WVP after the incorporation of ethanolic extract of propolis was reported in previous studies in different polymeric matrices, such as starch [101] and chitosan [102]. However, the presence of green propolis extract may have increased the free spaces in the polymer matrix, increasing the passage of vapors and inhibiting water absorption, resulting in an increase in WVTR and WVP, as was observed using the SEM technique (Figure 3b,c). This behavior was similar to that reported by Suriyatem et al. [103], where the presence of ethanolic extract of propolis resulted in increased WVP in carboxymethylchitosan films. Furthermore, it is important to note the WVTR and WVP results for sample F6 was significantly different when compared to pure BC, with an approximate increase of 243.84% and 290.39%, respectively. These results may be associated with the presence of hydrophilic regions of this wound dressing, also demonstrated in other characterization analyses in Figure 6.

### 3.4. Characterization of Active and Pure BC Wound Dressings: Mechanical Properties of Traction

**Figure 8:**
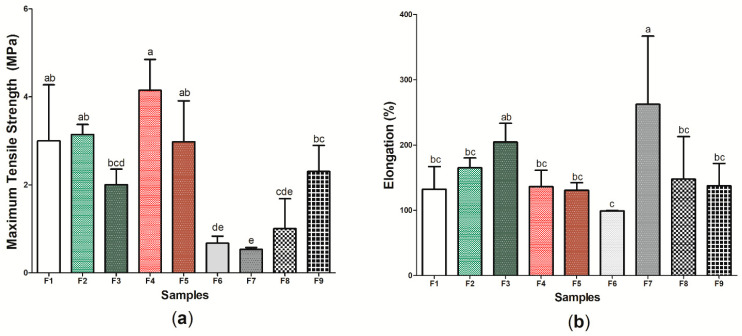
Tensile mechanical properties of wound dressings F1 to F9: (**a**) maximum tensile strength (Mpa) and (**b**) elongation (%). Bars followed by the same letters ^(a,b,c,d,e)^ were not significantly different considering <0.05 as *p* value according to Tukey’s test with 95% confidence level.

Figure 8 presents the results referring to the tensile mechanical properties of the nine (9) samples produced, characterized by the maximum tensile strength test (Figure 8a) and elongation test (Figure 8b) (Table S1). Sample F4 presented the highest maximum tensile strength (4.15 ± 0.701 MPa), while wound dressing F7 presented the lowest found value (0.672 ± 0.163 MPa), with a significant difference between the samples (*p* < 0.05). Similar to the results found in this study, Voss et al. [104] demonstrated that the incorporation of propolis extract into cellulose and polyvinyl acetate films did not significantly alter the maximum tensile strength values when compared to the control without the addition of the active substance. Studies have shown that BC has a tensile strength similar to human skin, which allows tissue regeneration to occur in a satisfactory manner, mainly by allowing the growth and migration of keratinocytes in vitro [105,106]. As such, samples F2, F3, F4, F5, and F9, which showed the values for this property with no significant difference (*p* > 0.05) to sample F1, may present a greater potential application for dermal epidermal lesions [107]. 

However, considering elongation (%), sample F7 had the highest result: 262.91 ± 104.309%. Importantly, the other active wound dressings (samples F2–F6, F8, and F9) did not show a significant difference when compared to the wound dressing formed only by BC (F1 sample), indicating that the increased concentration of *p*-coumaric acid in the cellulosic matrix had a plasticizing characteristic, increasing the mobility of its polymer chain and, consequently, the elongation of the material. The study by Contardi et al. [108] also demonstrated the increase in elongation from increasing *p*-coumaric acid concentration in PVP films, where the maximum value found was 300%. The use of wound dressings with high elongation may be important for the treatment of injuries that require constant applications and handling [109,110], thus indicating the potential application of wound dressings within this perspective.

### 3.5. Characterization of the Active and the Pure BC Wound Dressings: Flavonoids Content, Phenolic Compounds, Antioxidant, and Antimicrobial Activities

**Figure 9:**
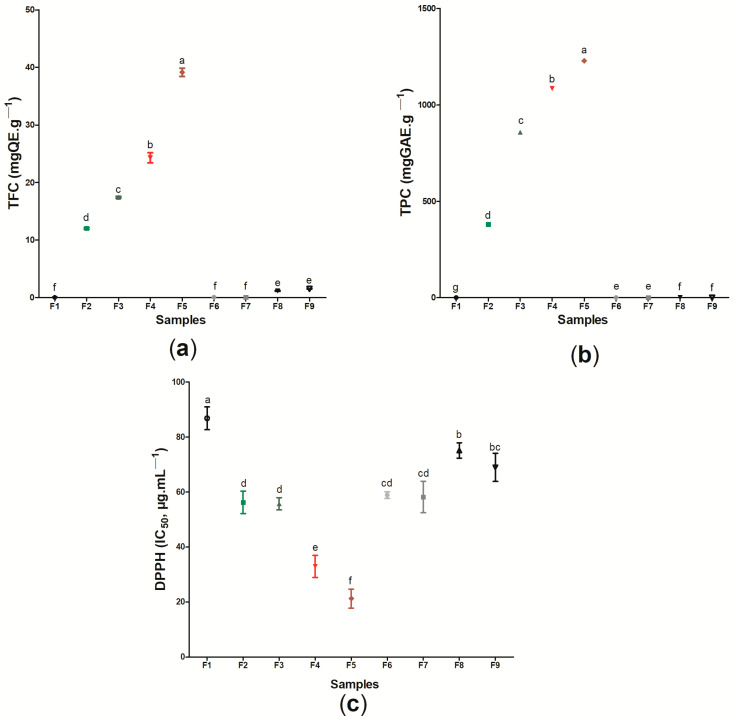
Determination of (**a**) TFC (mgEQ g^−1^), (**b**) TPC (mgGAE g^−1^), and (**c**) antioxidant activity (DPPH-IC_50_ μg mL^−1^). Values showing the same letter ^(a,b,c,d,e,f)^ in the same analysis do not show significant differences (*p* > 0.05) based on Tukey test at 95% confidence level.

Figure 9 presents the results regarding the TFC (Figure 9a), TPC (Figure 9b), and the antioxidant action based on the DPPH radical scavenging capacity (Figure 9c) of the sample F1 (pure BC) and the active wound dressings (samples F2–F9) (Table S1). In general, the results obtained showed that the TFC is related to the type and concentration of the active ingredient in the cellulose matrix. The low TFC of samples F6 and F7 formed by BC and *p*-coumaric acid (0.072 ± 0.004 mgQE g^−1^ and 0.0072 ± 0.001 mgQE g^−1^, respectively) is expected, since this substance does not belong to the flavonoid class, being a representative of phenolic acids–specifically, the hydroxycinnamic acid family [111]. Contrarily, biochanin A is considered an isoflavone of the flavonoid group, and the incorporation of 8 or 16 mg (samples F8 and F9, respectively) of this substance into the BC membrane was enough to promote a significant difference to the control (sample F1) [112]. Samples F4 and F5 were the samples that had the highest TFC, 24.3 ± 0.871 mgQE g^−1^ and 39.17 ± 0.723 mgQE g^−1^, respectively, followed by wound dressing F3 and wound dressing F2, with 17.43 ± 0.153 mgQE g^−1^ and 12.03 ± 0.152 mgQE g^−1^, respectively. 

Similar behavior was found for the analysis of total phenolic compounds, where the samples containing the ethanolic extract of red propolis were the ones that presented the highest values for this property (1084.10 mgGAE g^−1^ and 1228.33 mgGAE g^−1^ for samples F4 and F5, respectively), while sample F1 did not present any phenolic compounds in its composition. Different values for TFC and TPC among ethanolic extracts of distinct types of propolis have already been reported by other authors, where this behavior is commonly associated with geographic and climatic variations from where these samples are obtained [52,113,114,115]. The presence of phenolic compounds, including flavonoid compounds, during the healing process may result in the control of inflammatory activity. The study by Corrêa et al. [116] demonstrated that the use of the ethanolic extract of red propolis in mice promoted improved healing of skin lesions mainly due to the high concentration of phenolic compounds in its constitution, since these components can act by regulating the expression of the inflammatory transcription factor protein NF-κB (nuclear factor kappa B) and in reducing the production of inflammatory cytokines.

The flavonoid and phenolic contents may be related to different biological activities of wound dressings, including antioxidant activity and antimicrobial action. In the case of this study, the antioxidant activity of the wound dressings was based on the sequestration of the DPPH radical, in which a lower IC_50_ (µg mL^−1^) represents a higher antioxidant activity. When compared to sample F1, based only on BC, the eight (8) samples formed from the incorporation of active substances (ethanolic extracts of propolis or its biomarkers) showed a significant difference for this analysis (*p* < 0.05). The results found ranged from 21.23 ± 3.453 µg mL^−1^ (sample F5) to 86.77 ± 4.152 µg mL^−1^ (sample F1). The presence of propolis extract in different polymeric matrices, such as polycaprolactone (PCL) and cellulose acetate [117], pectin [73], and chitosan [102], caused these materials to show antioxidant action based on the ability to sequester the DPPH radical, where the increase of this activity was proportional to the increase in the amount of extract. The antioxidant action of propolis has been previously reported, being attributed mainly to the presence of phenolic compounds in its composition [32,118,119]. 

Importantly, the wound dressings formed from the incorporation of the green and red propolis biomarkers evaluated in this study, *p*-coumaric acid (samples F6 and F7) and biochanin A (samples F8 and F9), also showed antioxidant activity when compared to pure BC, with 58.9 ± 1.212 µg mL^−1^ for sample F5, 58.20 ± 5.730 µg mL^−1^ for sample F6, 75.13 ± 2.743 µg mL^−1^ for sample F8, and 68.96 ± 5.131 µg mL^−1^ for sample F9 (*p* < 0.05). Contardi et al. [74] reported that the DPPH radical scavenging capacity in ε-caprolactone and *p*-coumaric acid films increased over time, and could reach more than 90% after 180 min of exposure. Isolated biochanin A also demonstrated antioxidant activity in the study by Xue et al. [120], where the concentration of 3 to 15 mM was able to sequester up to 78% of the DPPH radical. Considering the use of these materials as dressings for injuries, it is noteworthy that the antioxidant action in this context can act to control inflammation by regulating inflammatory factors through the neutralization of reactive oxygen/nitrogen species, assisting in the healing process [121,122]. Thus, our results indicated that the use of the active wound dressings (samples F2–F9), especially those formed by BC and red propolis extract, can provide a better antioxidant action against reactive species after the incorporation of the analyzed active substances, which may be important for the healing process of lesions.

Regarding the antimicrobial activity, the *E. coli* (ATCC 8739) showed resistance to all the wound dressings developed, regardless of the active substance that was incorporated into the cellulosic matrix (Table 2 and Figure S20). However, considering the action against *S. aureus* (ATCC 6538), wound dressings F4 and F5 containing ethanolic extract of red propolis showed a zone of inhibition of 14.0 ± 1.00 mm and 18.0 ± 1.527 mm, respectively, with a significant difference (*p* < 0.05). Different authors have demonstrated that the ethanolic extract of red propolis shows a lower minimum inhibitory concentration when compared to the green propolis extract for Gram-positive bacterial species, which indicates a more effective antimicrobial activity of red propolis within this context, which was also found in this article [48,52,123]. Considering the incorporation of propolis extracts in polymeric matrices, Barud et al. [70] found results in which samples formed by BC membranes with ethanolic extract of propolis at concentrations of 1.2, 2.4 or 3.6% were able to form a zone of inhibition of bacterial growth between 7 and 10 mm against different species of *Staphylococcus*–smaller extensions than the one found in this work. 

On the other hand, Mocanu et al. [124] found similar results to our study, where films formed by BC, ethanolic extract of propolis, and zinc oxide (ZnO) showed no action against *E. coli*. Other studies show that propolis has a more effective antimicrobial activity against Gram-positive bacteria when compared to Gram-negative bacteria [125,126]. Among the reasons that may explain this behavior is the difference between the cell wall and membrane structure of these microorganisms, as well as the possible production of hydrolytic enzymes among Gram-negative bacteria that are able to inactivate the active components of propolis [127,128]. Importantly, samples F8 and F9 based on BC and red propolis biomarkers (biochanin A) did not show antimicrobial activity against *S. aureus*, unlike samples containing the ethanolic extract of red propolis as an active substance (samples F4 and F5), which may indicate the importance of the synergistic effect between the components of propolis, as well as the need to adjust the concentration of the active substance in the polymeric matrix. It is also noteworthy that *S. aureus* bacteria are known for their ease in colonizing the bed of lesions and, through the secretion of proteases and toxins and the formation of biofilms, have been responsible for hindering the healing process [129,130]. Thus, our results suggest that samples F4 and F5 may represent an alternative for the treatment of lesions due to their action against *S. aureus*.

### 3.6. Multivariate Statistical Analysis of Active and Pure BC Wound Dressings

**Figure 10:**
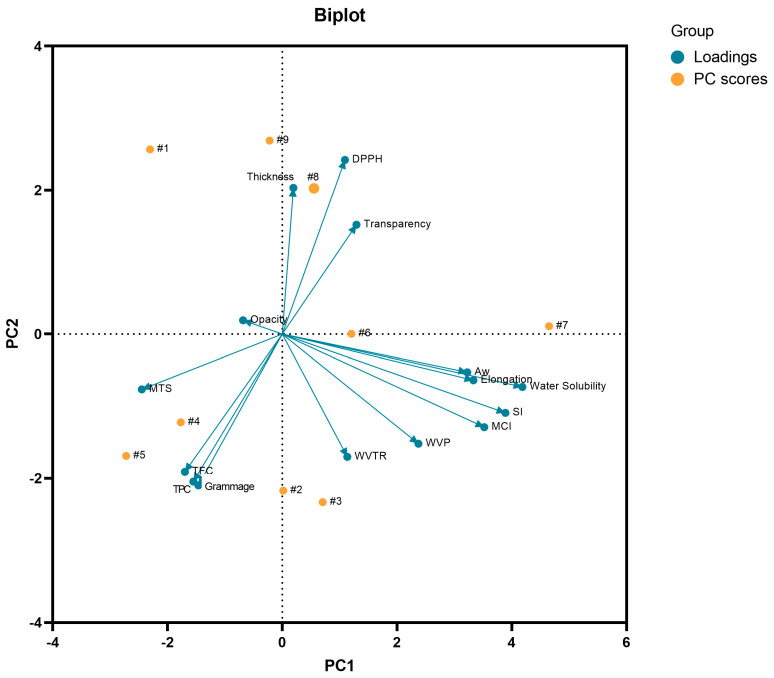
Biplot analysis of PC1 × PC2 considering the PC scores (samples F1 to F9) and the characterization variables (loadings) considered for the PCA. MCI: moisture content index; MTS: maximum tensile strength; SI: swelling index: TFC: total flavonoid content; TPC: total phenolic content; WVP: water vapor permeability; WVPR: water vapor permeability rate.

The principal component analysis (PCA) was performed in order to evaluate the relationship between the investigated properties (opacity, transparency, aw, swelling index, moisture content index, water solubility, thickness, grammage, WVPR, elongation, WPV, DPPH radical scavenging capacity, TFC, and TPC) and the components of the nine developed wound dressings (Figure 10). It is important to highlight that PCA is an advanced multivariate statistical technique, where its basic principle is to act in reducing the dimension of large data sets, thus increasing the interpretability of these data sets with minimal loss of information [131].

In this study, PC1 explained 33.06% of the total variance of the data, while PC2 explained 27.16%, thus explaining 60.22% of the cumulative variance on the optical, mechanical, biological, and physical and barrier properties of the wound dressings, where water solubility had the highest weight in PC1 and grammage had the highest weight in PC2. In general, PCA also showed that the samples tended to group according to the active substance incorporated in the BC matrix. From this analysis, it was shown that the sample F1 was not grouped with any of the active wound dressings (samples F2–F9). In addition, it is noteworthy that the wound dressings F6 and F7 formed by BC and 8 mg or 16 mg of *p*-coumaric acid, respectively, were allocated in different quadrants, which was influenced by the variables (loadings) of water solubility, aw, swelling index, elongation, and moisture content index. This behavior was demonstrated in Figure 6, where the higher concentration of *p*-coumaric acid in the BC resulted in a wound dressing with a more hydrophilic character. It is also noteworthy that some variables showed a negative correlation with each other, such as the content of flavonoids and phenolic compounds with the ability to scavenge the DPPH radical, as shown in Figure 10, where the samples with the highest content of flavonoids and phenolic compounds showed a lower IC_50_ related to the ability to scavenge the DPPH radical. Therefore, multivariate analysis, more specifically PCA, demonstrated that the type and concentration of the active substance in the BC matrix influenced the optical, physical, barrier, mechanical, and biological properties evaluated. 

### 3.7. In Vitro Cutaneous Permeation and Retention of Active Wound Dressings

**Figure 11:**
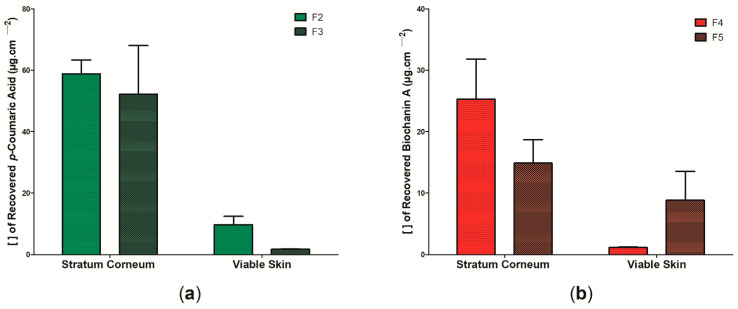
Concentration of propolis biomarkers in the stratum corneum and viable skin in the in vitro permeation assay on pig skin after 2 h of passive application: (**a**) permeation of wound dressings F2 and F3 according to *p*-coumaric acid concentration and (**b**) permeation of wound dressings F4 and F5 according to biochanin A concentration. Bars represent the mean ± standard deviation of the mean of 4 determinations.

The ability of the active substances to be released from the polymeric matrix of the wound dressings and to penetrate the skin layers was assessed using Franz cell diffusion. The *p*-coumaric acid present in samples F6 and F7 and the biochanin A present in samples F8 and F9 were not retained in any of the skin layers analyzed in this study, suggesting that the evaluated system (BC: propolis biomarkers) may present some kind of instability. Thus, Figure 11 shows the results regarding the cutaneous permeation in pig skin of wound dressings F2, F3, F4, and F5 from the analysis of the recovery of *p*-coumaric acid biomarkers (Figure 11a), for the wound dressings formed by BC and ethanolic extract of green propolis (samples F2 and F3) and biochanin A (Figure 11b), and for the wound dressings formed by BC and ethanolic extract of red propolis (samples F4 and F5). There was no significant difference by the Bonferroni post-test performed. In general, the results showed that the concentration of biomarkers, regardless of the wound dressing, was higher in the stratum corneum than in the viable skin. Sample F2 showed the highest retention of biomarker (*p*-coumaric acid) after 2 h of exposure, where in the stratum corneum the retention was ~603% higher than in the viable skin, with 58.79 ± 9.062 µg cm^−2^ and 9.74 ± 5.405 µg cm^−2^, respectively. The retention of *p*-coumaric acid from the permeability analysis of sample F3 was 52.15 ± 31.75 µg cm^−2^ for the stratum corneum and 1.746 ± 0.2 for the viable skin. 

The study by Dias et al. [132] evaluated the retention of biochanin A in the stratum corneum and viable skin after passive permeation for 8 h, where the results found were 0.28 and 0.54 µg cm^−2^, respectively; these are lower values than the ones found in this study for the wound dressings containing red propolis extract (sample F4 and F5) and that were also evaluated from the concentration of this biomarker. The authors also reported that biochanin A showed better skin permeability than formononetin, another molecule belonging to the isoflavones group [132]. The study by dos Santos et al. [133], on the other hand, demonstrated that the ethanolic extract of green propolis showed a retention of 2.01 ± 0.85 µg cm^−2^ in the stratum corneum, with its removal allowing better permeation in other layers of the skin, such as the dermis. Marquele-Oliveira et al. [134] developed a BC-based membrane incorporated with a self-emulsifying propolis formulation, where the authors observed that the release of two biomarkers (*p*-coumaric acid and artepelin C) was faster in the first 24 h, with *p*-coumaric acid showing a faster release than artepelin C.

In this study, it was observed that the retention of *p*-coumaric acid in the stratum corneum and viable skin was higher than that of biochanin A during the 2 h of passive permeability. Furthermore, it is important to note that the stratum corneum represents the outermost layer of the skin and is mainly formed by horny scales and keratin, presenting a lipophilic character [135]. The retention of *p*-coumaric acid and biochanin A in this outermost layer may be the result of lipophilic interactions of these molecules already reported in previous studies [132,136]. Importantly, *p*-coumaric acid has an amphiphilic character, meaning that it has both hydrophilic and hydrophobic properties, which may explain its hydrophilic profile reported in Figure 6 and Figure 11, as well as its possible interaction with the molecules in the stratum corneum [45]. The presence of active components in the stratum corneum may be an important alternative for promoting tissue recovery in superficial dermal lesions, as it can act to combat opportunistic pathogens that may hinder the healing process [137]. Within these perspectives, it is suggested that samples F2, F3, and F4 can be used for this purpose. However, sample F5 showed the highest retention of the biomarker in viable skin, which may be important for the treatment of deeper lesions.

## 4. Conclusions

The results found in the present study suggest that, in general, the active wound dressings formed through the incorporation of ethanolic extracts of green and red propolis, as well as their biomarkers–*p*-coumaric acid and biochanin A–in bacterial cellulose polymer matrices present distinct properties when compared to wound dressings based only on bacterial cellulose. The wound dressings containing *p*-coumaric acid (samples F6 and F7) showed a higher hydrophilic character when compared to the other samples. Samples F4 and F5, where the active substance was the ethanolic extract of red propolis, were the only ones that presented antimicrobial activity against *S. aureus*, which may be related to the high content of flavonoids and phenolic found and the high capacity of DPPH radical sequestration. Furthermore, the permeation and retention study demonstrated that the biomarkers of the wound dressings containing propolis extract (samples F2, F3, F4, and F5) are retained in a higher concentration in the stratum corneum, while the propolis biomarkers present in samples F6, F7, F8, and F9 were not quantified in any of the skin layers analyzed. Given the different performances, the application of each of the nine wound dressings developed may be related to the type of dermal injury to be treated as well as the stage of healing, which may be an important alternative for the modern treatment of dermal injuries.

## Figures and Tables

**Table 1:** Experimental design of the BC-based wound dressing with added active compounds.

Name of Sample	Composition of Wound Dressing (%, m·v^−1^)				
	BC	EtOH Green	EtOH Red	*p*-Coumaric Acid	Biochanin A
F1	50	-	-	-	-
F2	50	2	-	-	-
F3	50	4	-	-	-
F4	50	-	2	-	-
F5	50	-	4	-	-
F6	50	-	-	8	-
F7	50	-	-	16	-
F8	50	-	-	-	8
F9	50	-	-	-	16

EtOH: Ethanolic extract.

**Table 2:** Mean ± standard deviation of the diameter (mm) of the zone of inhibition of wound dressings F1 to F9 against *Escherichia coli* (ATCC 8739) and *Staphylococcus aureus* (ATCC 6538).

Wound Dressings (Samples)	Inhibition Zone Diameter (mm)	
	*Staphylococcus aureus* (ATCC 6538)	*Escherichia coli* (ATCC 8739)
F1	R	R
F2	R	R
F3	R	R
F4	14.0 ± 1.00 ^b^	R
F5	18.0 ± 1.527 ^a^	R
F6	R	R
F7	R	R
F8	R	R
F9	R	R

R: resistant. Values with the same superscript letter ^(a, b)^ in the same column (*p* > 0.05) showed no significant difference between, according to Tukey’s test with 95% confidence level.

**Figure A1:**
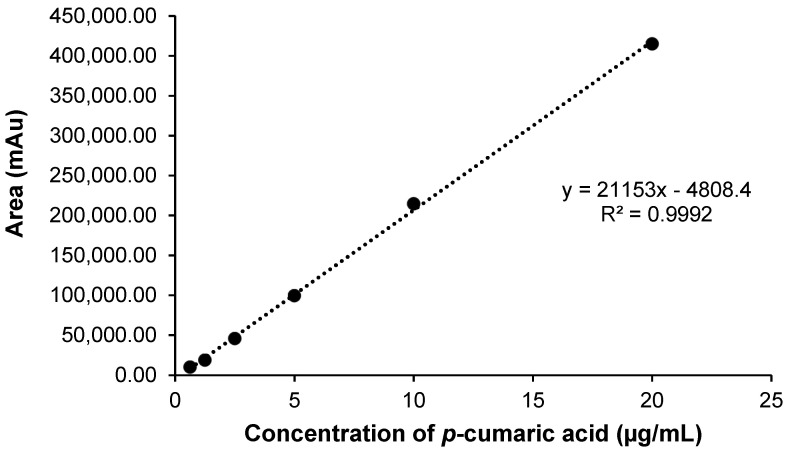
Graphical representation of the analytical curve obtained for *p*-coumaric acid by HPLC. Concentrations diluted in methanol. Equation of the straight line: y = 21,153x − 4808.4 and linear correlation coefficient: r = 0.9992.

**Figure A2:**
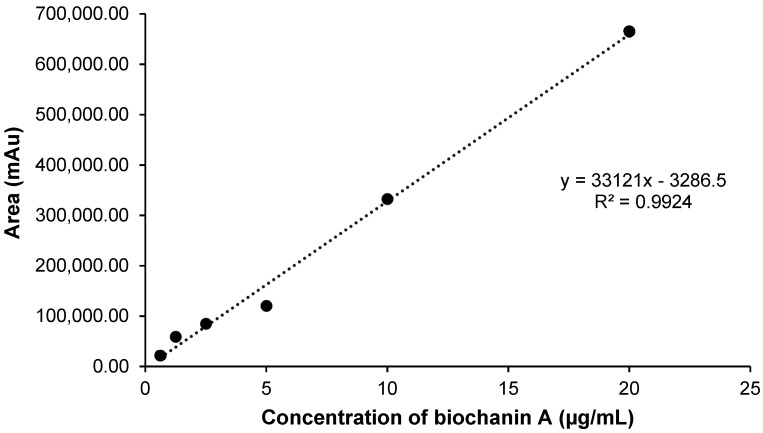
Graphical representation of the analytical curve obtained for biochanin A by HPLC. Concentrations diluted in methanol. Equation of the straight line: y = 33,121x − 3286.5 and linear correlation coefficient: r = 0.9924.

## Data Availability

Not applicable.

## Supplementary Materials

The following supporting information can be download at: https://www.mdpi.com/article/10.3390/pharmaceutics14061222/s1, Figure S1: (a) Bacterial cellulose production kinetics determined from bacterial growth (OD_Abs500 nm_) and total soluble solids content (°Brix) during 14 days of static culture; (b) Purified cellulose membrane after alkaline treatment; Figure S2: Original image of wound dressing F1; Figure S3: Original image of wound dressing F2; Figure S4: Original image of wound dressing F3; Figure S5: Original image of wound dressing F4; Figure S6: Original image of wound dressing F5; Figure S7: Original image of wound dressing F6; Figure S8: Original image of wound dressing F7; Figure S9: Original image of wound dressing F8; Figure S10: Original image of wound dressing F9; Figure S11: Original format of the scanning electron microscopy surface micrograph of the surface of the F1 wound dressing; Figure S12: Original format of the scanning electron microscopy surface micrograph of the surface of the F2 wound dressing; Figure S13: Original format of the scanning electron microscopy surface micrograph of the surface of the F3 wound dressing; Figure S14: Original format of the scanning electron microscopy surface micrograph of the surface of the F4 wound dressing; Figure S15: Original format of the scanning electron microscopy surface micrograph of the surface of the F5 wound dressing; Figure S16: Original format of the scanning electron microscopy surface micrograph of the surface of the F6 wound dressing; Figure S17: Original format of the scanning electron microscopy surface micrograph of the surface of the F7 wound dressing; Figure S18: Original format of the scanning electron microscopy surface micrograph of the surface of the F8 wound dressing; Figure S19: Original format of the scanning electron microscopy surface micrograph of the surface of the F9 wound dressing; Figure S20: Analysis of the antimicrobial activity of the 8 active wound dressings developed (F2, F3. F4, F5, F6, F7, F8 and F9, referring to letters A, B, C, D, E, F, G and H, respectively): against (a) *E. coli* and (b) *S. aureus*; Table S1: Optical, physical, barrier, and mechanical properties, as well asTFC, TPC and antioxidant activity of nine wound dressings based on bacterial cellulose and different active substances (ethanolic extract of green and red propolis, *p*-coumaric acid and biochanin A). No significant difference between values with the same superscript letter ^(a,b,c,d)^ in the same row (*p* > 0.05), according to the Tukey test with 95% confidence.

## Appendix A

### Appendix A.1. Analytical Method for the Quantification of p-Coumaric Acid and Biochanin A

The *p*-coumaric acid and biochanin A were measured using a Shimadzu LC 20-AD model HPLC, consisting of two pumps (model LC 20-AT), an automatic injector (model 9SIL-20AD) and oven (model CTO-20AS), coupled to a spectrophotometric detector (model SPD-M20A) and a computer equipped with the Shimadzu LC chromatographic analysis program. A C18 reversed phase column (150 mm × 4.6 mm) and mobile phase composed of a mixture of ultrapurified water:acetronitrile (90:10) (*v*/*v*) were used. The flow rate was 1 mL min^−1^, the injection volume of the samples was 50 µL, the oven was used at room temperature, and detection by Diodes Arrangement Detector (DAD) was performed at a wavelength of 245 nm for *p*-coumaric acid and 340 nm for biochanin A.

### Appendix A.2. Validation of the Methods

The standardized methods were validated in terms of linearity, specificity, and selectivity and limits of detection and quantification against skin interferents for use in skin permeation assays.

#### Appendix A.2.1. Linearity

To check the linearity of the method, six dilutions in methanol were performed from a standard solution of the markers *p*-coumaric acid and biochanin A (100 µg mL^−1^) (HPLC grade, Tedia, Brazil). Dilutions to construct the curve were made in triplicate for each concentration, which were equal to 0.625; 1.25; 2.5; 5.0; 10.0 and 20.0 µg mL^−1^. The analytical curve was constructed by relating the concentration of the markers with the areas of the peaks provided by the apparatus after injection of the standard samples. The statistical analysis of the data was obtained by the linear regression method, obtaining a straight line in the format y = ax + b, where (a) corresponds to the angular coefficient and (b) linear coefficient. The linear ranges were calculated using the linear correlation coefficient (r), which according to the minimum acceptable parameters of r = 0.99 [138].

#### Appendix A.2.2. Specificity or Selectivity

For the analysis of the specificity or selectivity of the method, methanol and a methanolic solution of the filtered pig ear skin homogenate were injected into the HPLC as sample blanks to verify the ability of the method to measure exactly one compound in the presence of other components [138].

#### Appendix A.2.3. Recovery Study of Markers (p-Coumaric Acid and Bichanin A) from Stratum Corneum and Viable Skin

Three fragments of pig skin were initially stretched and secured on a Styrofoam support. The stratum corneum of each of the skin pieces was removed by the tape stripping technique using 15 pieces of tape, which were placed in amber glass jars. The viable skins were cut into smaller fragments, which were placed in amber glass jars. Subsequently, on the pieces of tape containing the stratum corneum and the remaining skin pieces, 75 µL of the methanolic stock solution (100 µg mL^−1^) of the markers were added. The methanol was evaporated and then 5 mL of methanol was added for extraction of the markers from the skin layers and evaluation of recovery over a medium concentration range (1.5 µg mL^−1^) of the analytical curve. The vials were left unstirred for 24 h, and their contents were then filtered on 0.45 µm hydrophobic filter membranes connected to syringes and analyzed by HPLC for quantification of the markers. This experiment was performed in triplicate for each concentration analyzed.

### Appendix A.3. Results of the Analytical Method Standardization

#### Appendix A.3.1. Linearity

The linearity of the method corresponds to its ability to provide results directly proportional to the concentration of the analyte, within a range of application [139]. Figure A1 and Figure A2 show the analytical curves obtained for *p*-coumaric acid and bichanin A, respectively, in the concentration range from 0.625 to 20.0 µg mL^−1^. From the linear regression analysis it was possible to state that the methods showed linearity in the concentration range between 0.625 µg mL^−1^ and 20.0 µg mL^−1^, since the linear correlation coefficient obtained was 0.9999 for the methanol curve, being in accordance with that recommended by Anvisa (Agência Nacional de Vigilância Sanitária) [138].

#### Appendix A.3.2. Specificity or Selectivity

The specificity or selectivity of a method is its ability to exactly measure one compound in the presence of other components (e.g., impurities, degradation products, and matrix components) [138]. The retention times of the marker samples containing the skin filtrate did not overlap with those of the markers, showing the ability of the method to quantify the markers reliably.

#### Appendix A.3.3. Recovery

The extraction process of *p*-coumaric acid and biochanin A from the skin layers proved efficient for extraction of the markers within the linearity range (0.625 to 20.0 µg mL^−1^) of the analytical method validated by HPLC-UV. Table A1 and Table A2 shows the recovery results of *p*-coumaric acid and biochanin A extracted with 5 mL of methanol from stratum corneum and viable skin, respectively.

**Table A1:** Recovery values in % of *p*-coumaric acid from stratum corneum and viable skin.

Concentration of *p*-Coumaric Acid (µg mL^−1^)	Theoretical Concentration (µg mL^−1^)	Recovery Concentration (µg mL^−1^)	% Recovered Recovered of *p*-Coumaric Acid
Stratum corneum	1.75	1.69 ± 0.32	96.66 ± 0.32
Viable skin	1.75	1.71 ± 0.01	98.04 ± 0.01

**Table A2:** Recovery values in % of biochanin A acid from stratum corneum and viable skin.

Concentration of Biochanin A (µg mL^−1^)	Theoretical Concentration (µg mL^−1^)	Recovery Concentration (µg mL^−1^)	% Recovered Recovered of *p*-Coumaric Acid
Stratum corneum	1.75	1.77 ± 0.37	102.42 ± 0.37
Viable skin	1.75	1.70 ± 0.01	97.99 ± 0.01

The recovery of the markers was performed with error percentages that are within the accepted limit for the validation of methods involving extraction processes (±15%). This ensures that the standardized methodology is suitable for quantification of the markers present in the stratum corneum and viable skin.
