# Supplementary material for: Active Potential of Bacterial Cellulose-Based Wound Dressing: Analysis of Its Potential for Dermal Lesion Treatment
Source: Pharmaceutics. 2022 Jun 8;14(6):1222. doi: 10.3390/pharmaceutics14061222 (PMC9228207; doi:10.3390/pharmaceutics14061222)
Supplement: Supplementary file 1 [file pharmaceutics-14-01222-s001.zip › pharmaceutics-1741012-supplementary.pdf]

Supplementary Material

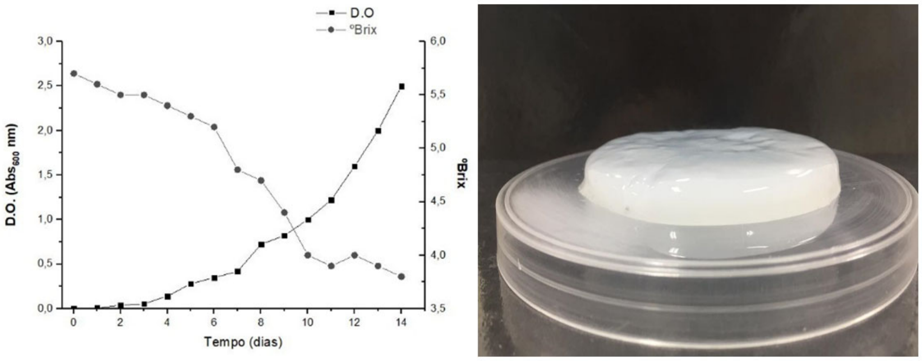

(a) (b)

Figure S1. (**a**) Bacterial cellulose production kinetics determined from bacterial growth ($OD_{Abs500nm}$) and total soluble solids content (ºBrix) during 14 days of static culture; (**b**) Purified cellulose membrane after alkaline treatment.

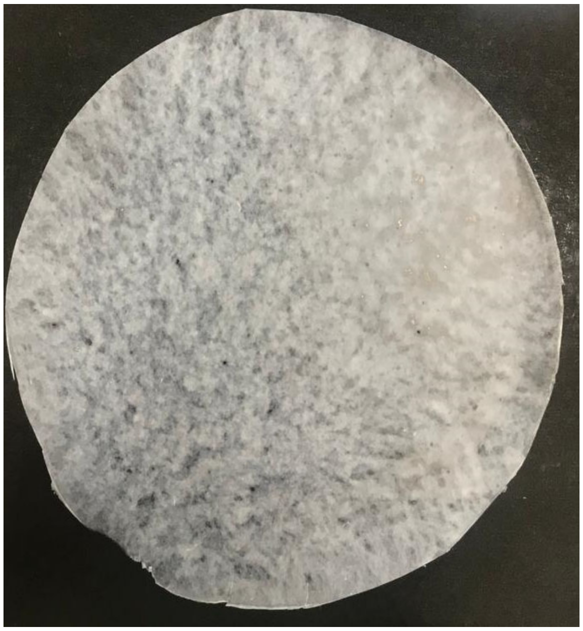

Figure S2. Original image of wound dressing F1.

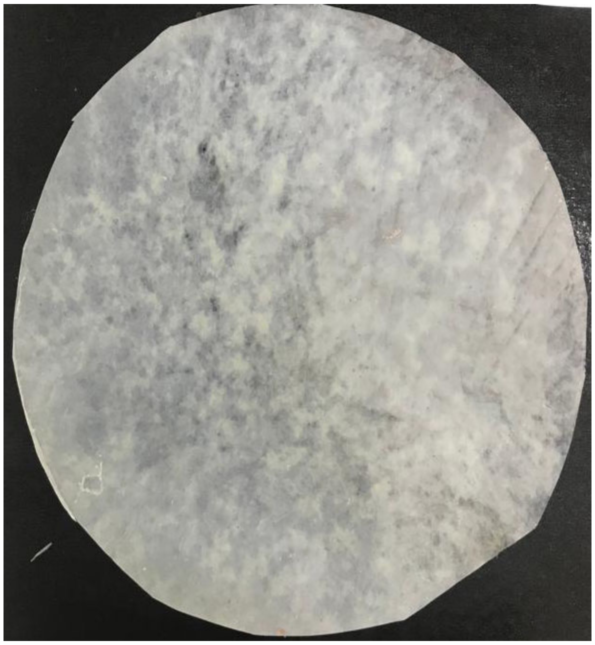

Figure S3. Original image of wound dressing F2.

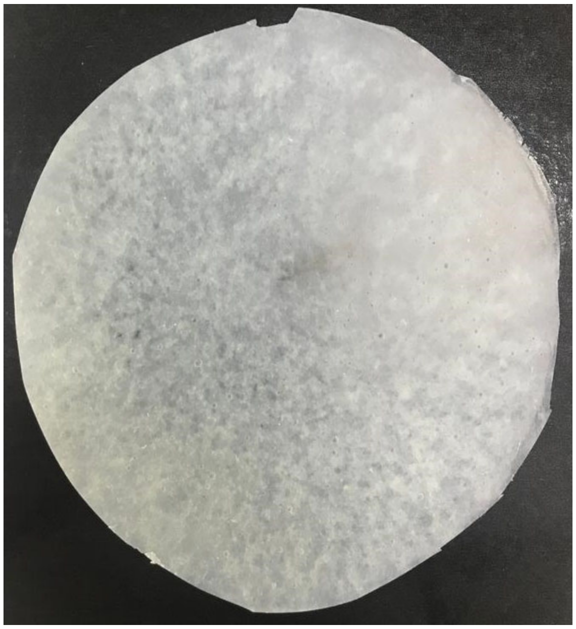

Figure S4. Original image of wound dressing F3.

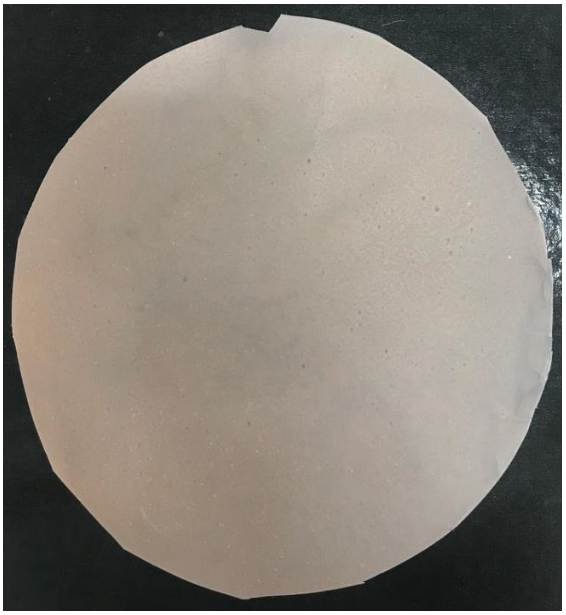

Figure S5. Original image of wound dressing F4.

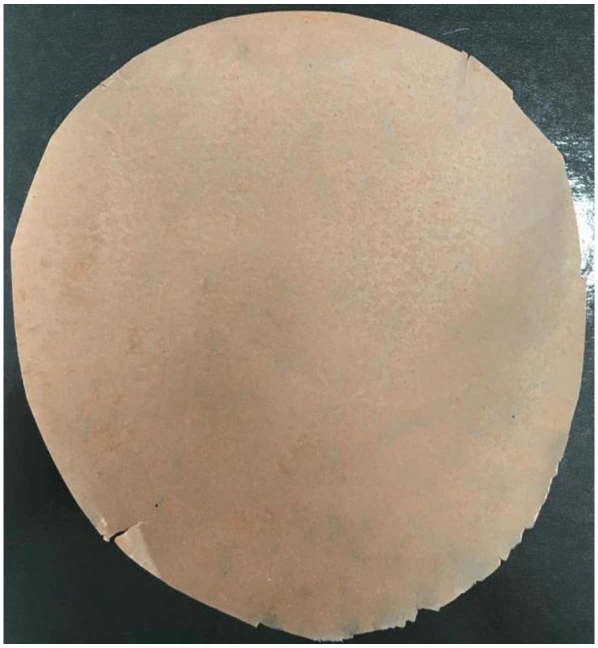

Figure S6. Original image of wound dressing F5.

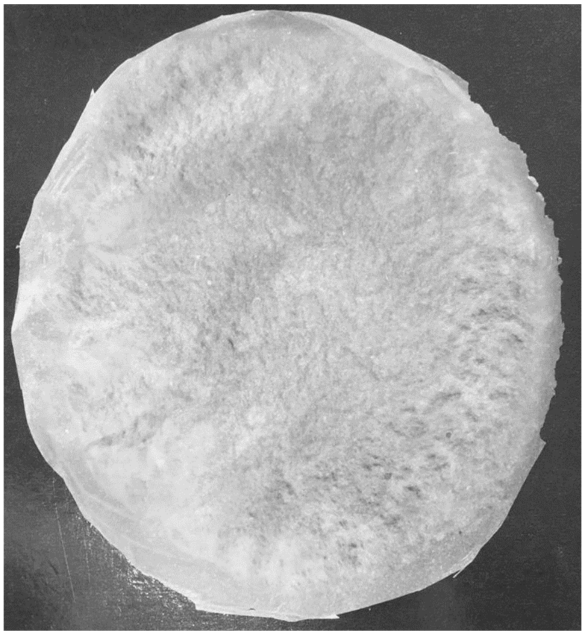

Figure S7. Original image of wound dressing F6.

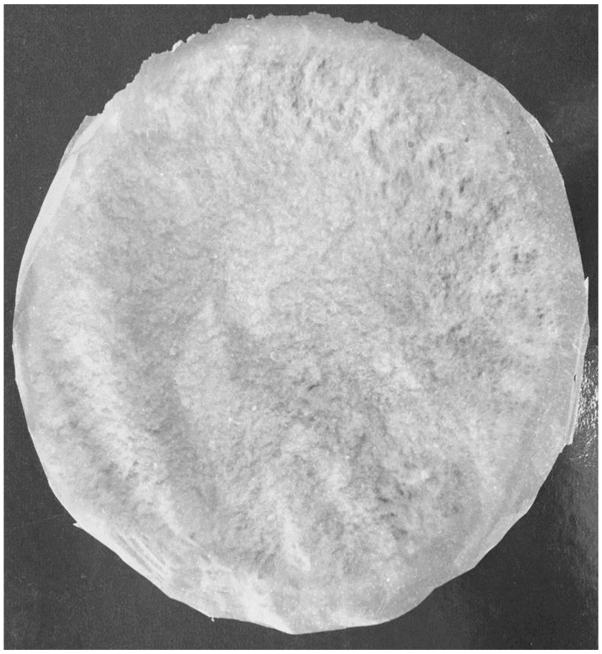

Figure S8. Original image of wound dressing F7.

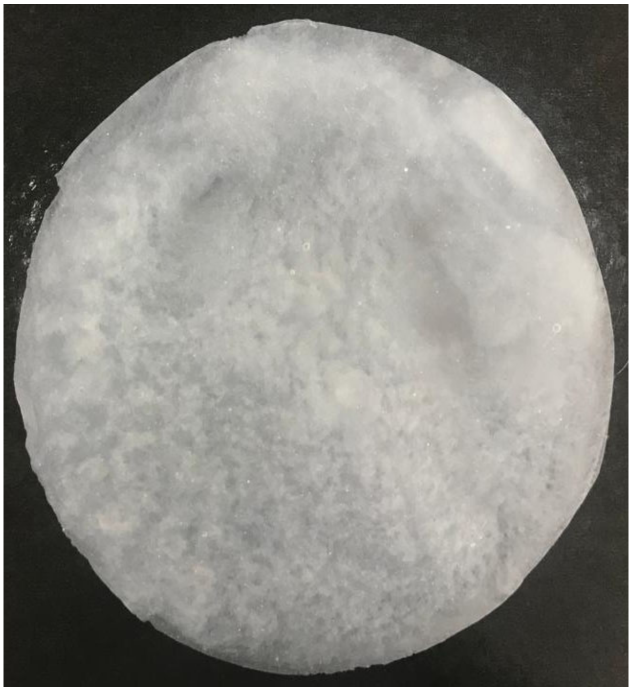

Figure S9. Original image of wound dressing F8.

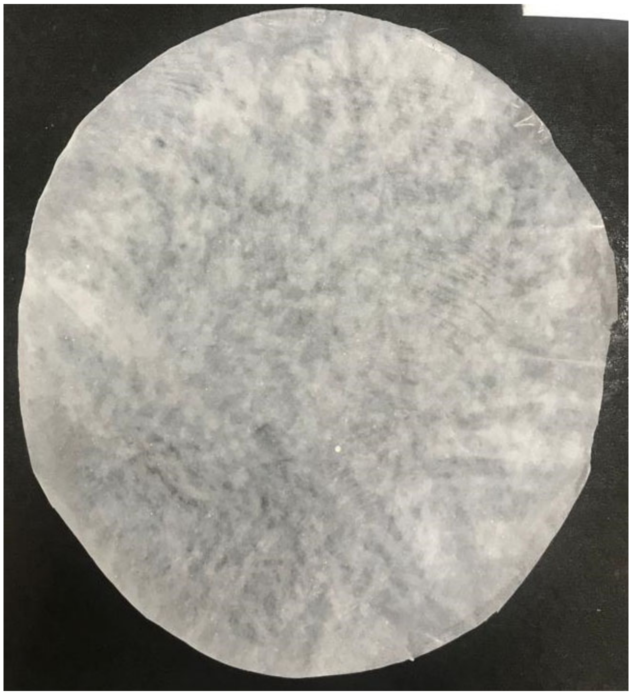

Figure S10. Original image of wound dressing F9.

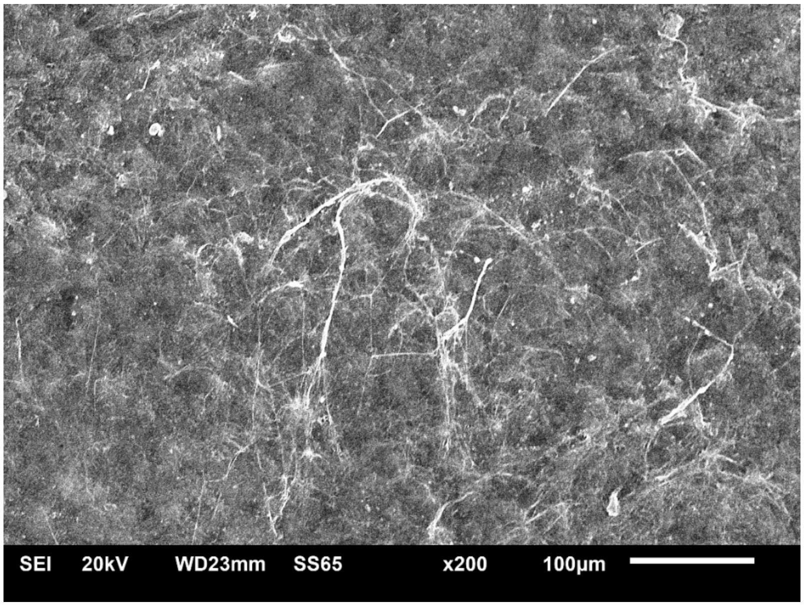

Figure S11. Original format of the scanning electron microscopy surface micrograph of the surface of the F1 wound dressing.

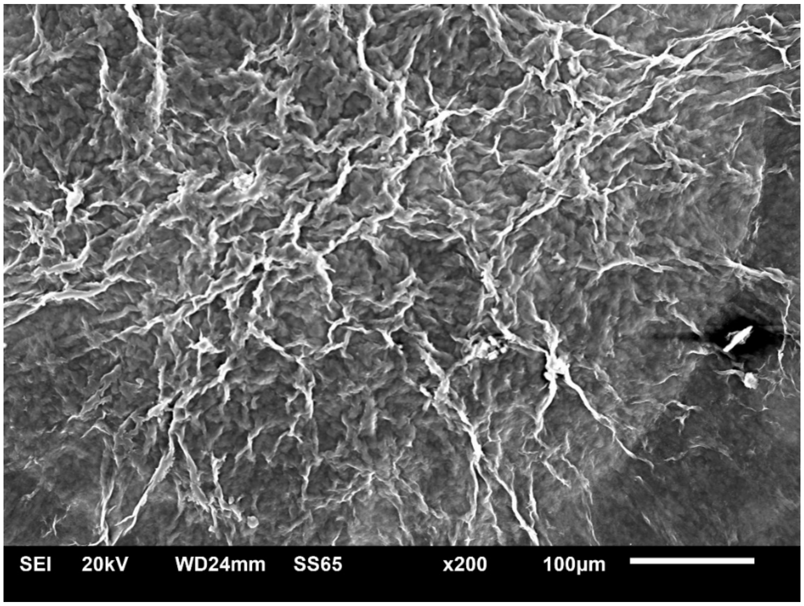

Figure S12. Original format of the scanning electron microscopy surface micrograph of the surface of the F2 wound dressing.

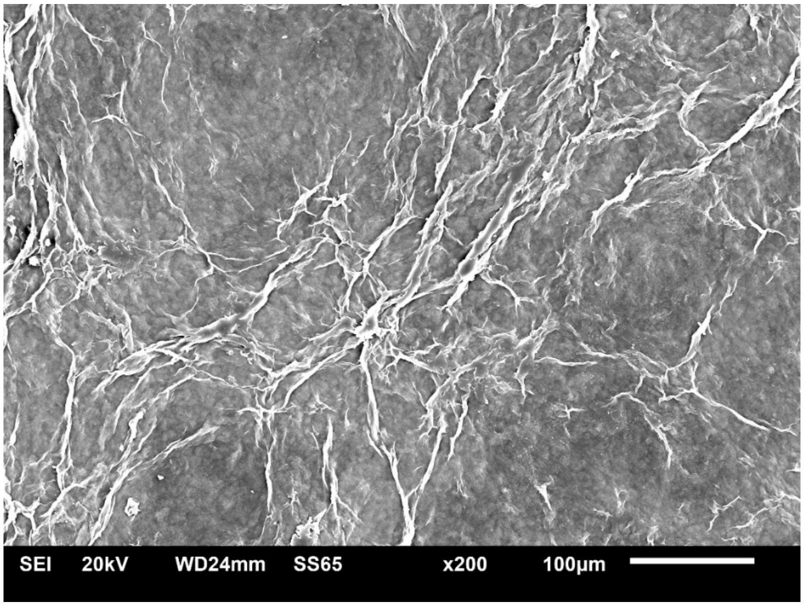

Figure S13. Original format of the scanning electron microscopy surface micrograph of the surface of the F3 wound dressing.

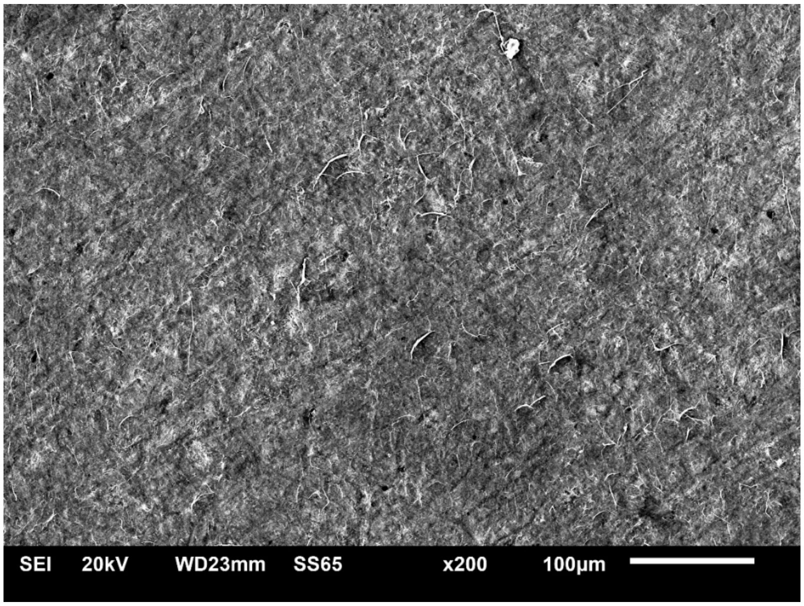

Figure S14. Original format of the scanning electron microscopy surface micrograph of the surface of the F4 wound dressing.

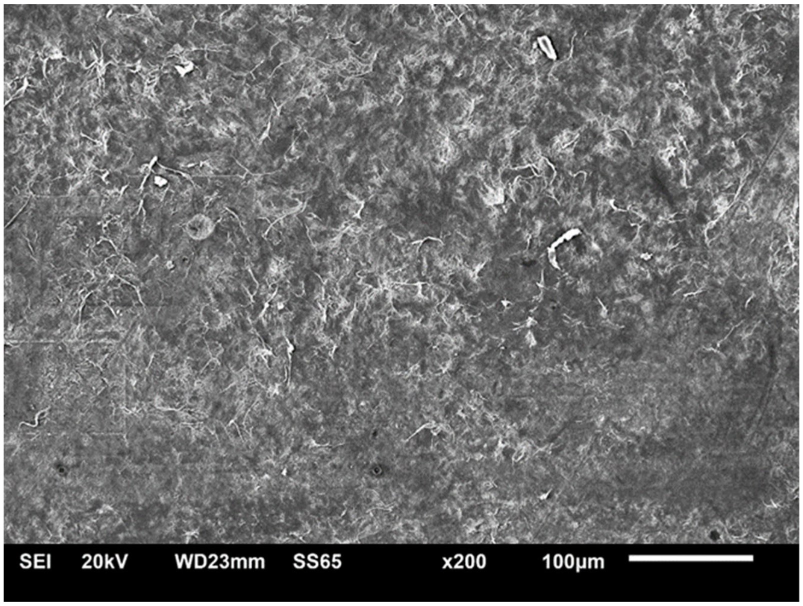

Figure S15. Original format of the scanning electron microscopy surface micrograph of the surface of the F5 wound dressing.

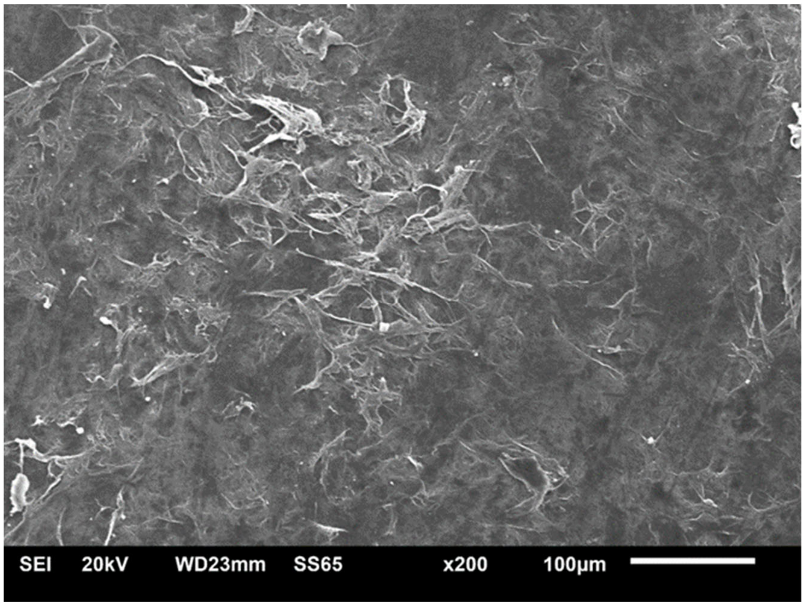

Figure S16. Original format of the scanning electron microscopy surface micrograph of the surface of the F6 wound dressing.

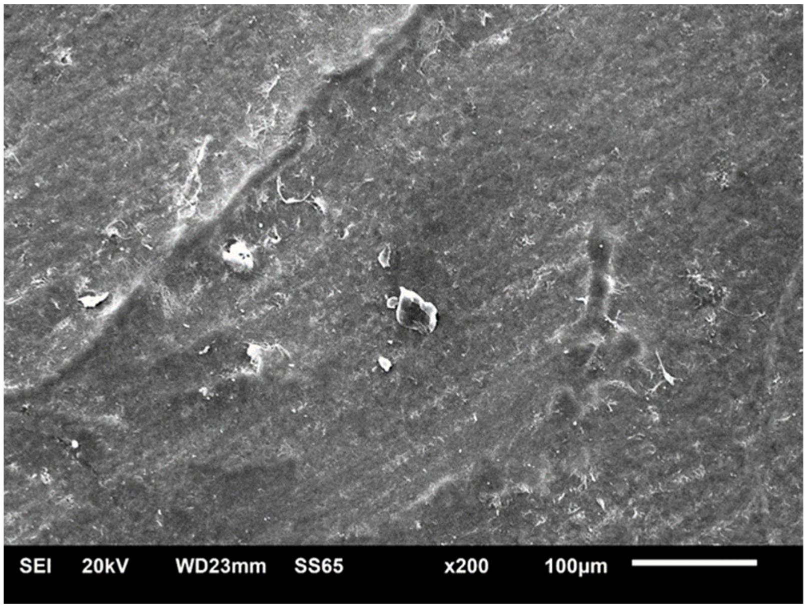

Figure S17. Original format of the scanning electron microscopy surface micrograph of the surface of the F7 wound dressing.

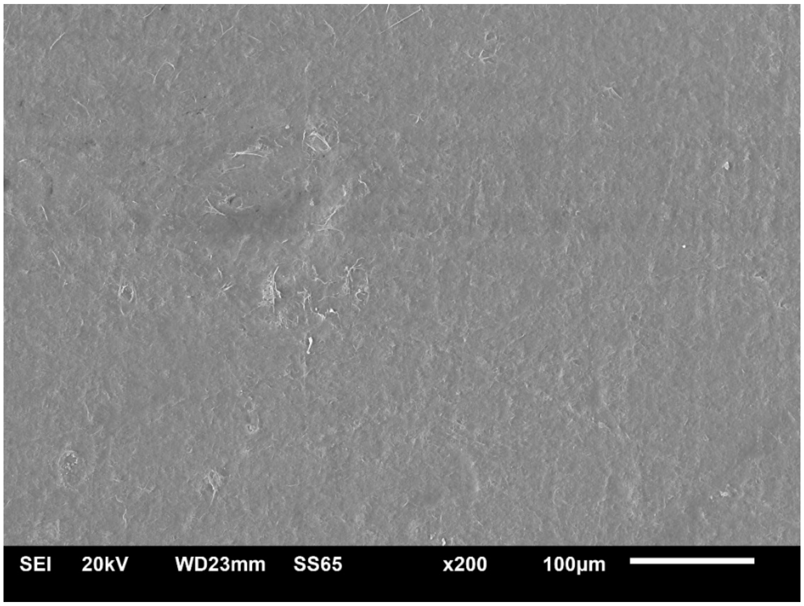

Figure S18. Original format of the scanning electron microscopy surface micrograph of the surface of the F8 wound dressing.

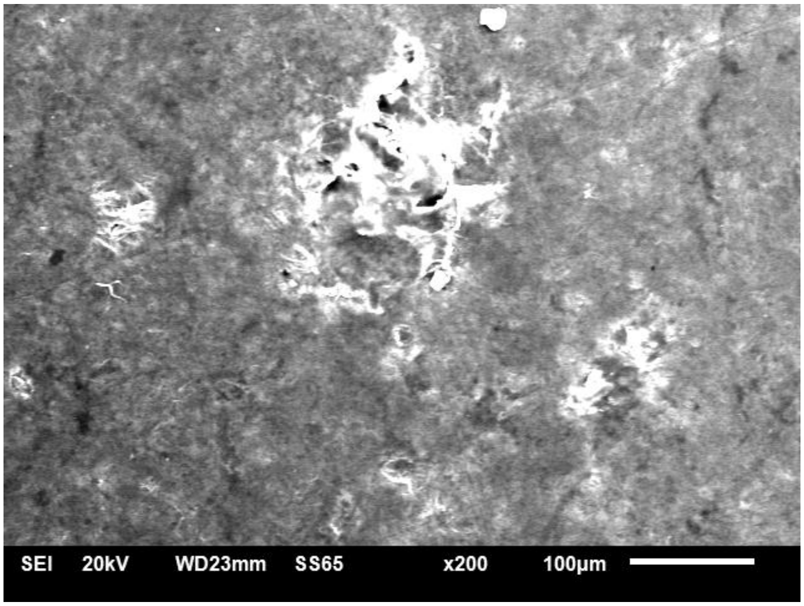

Figure S19. Original format of the scanning electron microscopy surface micrograph of the surface of the F9 wound dressing.

Table S1. Optical. Physical, barrier, and mechanical properties, as well as TFC, TPC and antioxidant activity of nine wound dressings based on bacterial cellulose and different active substances (ethanolic extract of green and red propolis, *p*-coumaric acid and biochanin A). No significant difference between values with the same superscript letter $^{(a,b,c,d)}$ in the same row ($p > 0.05$), according to the Tukey test with 95% confidence.

Tests	Samples								
	F1	F2	F3	F4	F5	F6	F7	F8	F9
Transparency (T_{600}.mm^{-1})	31.80±2.569^{e}	33.80±2.228^{de}	28.59±0.908^{e}	87.71±8.771^{ab}	58.43±9.237^{b}	53.99±8.861^{cd}	94.50±1.798^{c}	93.53±11.301^{ab}	110.62±12.077^{a}
Opacity (A_{500}.mm^{-1})	50.48±0.151^{a}	34.54±3.207^{bc}	39.45±1.004^{b}	29.85±1.171^{c}	41.63±2.947^{b}	42.40±6.381^{ab}	35.95±2.489^{bc}	34.31±2.817^{bc}	28.43±1.804^{c}
Grammage (g.cm^{-2})	0.006±0.001^{ab}	0.009±0.003^{a}	0.007±0.001^{ab}	0.008±0.001^{ab}	0.009±0.001^{a}	0.008±0.001^{ab}	0.004±0.001^{b}	0.006±0.001^{ab}	0.005±0.001^{b}
Thickness (mm)	0.029±0.001^{bc}	0.023±0.001^{c}	0.029±0.004^{bc}	0.024±0.001^{c}	0.034±0.006^{b}	0.029±0.004^{bc}	0.031±0.006^{b}	0.033±0.005^{b}	0.046±0.006^{a}
a_w	0.354±0.010^{bc}	0.344±0.012^{bcd}	0.360±0.001^{b}	0.324±0.001^{cde}	0.338±0.008^{bcd}	0.435±0.029^{a}	0.455±0.011^{a}	0.314±0.007^{de}	0.292±0.003^{e}
Water solubility (%)	6.25±0.000^{e}	32.89±7.292^{bcd}	33.89±7.978^{bc}	34.63±6.258^{bc}	11.04±1.204^{e}	44.49±6.969b	65.58±4.073^{a}	28.54±1.628^{cd}	18.97±1.012^{de}
Moisture Content Index (%)	27.44±10.819^{c}	70.32±3.576^{ab}	65.38±9.093^{ab}	62.75±3.179^{ab}	58.33±15.362^{ab}	66.97±0.755^{ab}	84.87±2.270^{a}	64.96±1.567^{ab}	56.36±2.421^{b}
Swelling Index (%)	48.93±6.020^{c}	240.52±43.694^{b}	234±12.222^{bc}	169.83±23.752^{bc}	202.03±10.184^{bc}	202.90±6.862^{bc}	405.55±4.811^{a}	185.85±12.962^{bc}	175.44±5.196^{bc}
WVTR	7.86±1.530^{c}	33.36±1.488^{a}	38.11±5.762^{a}	8.78±0.577^{c}	8.66±0.678^{c}	19.42±0.261^{b}	11.79±0.805^{c}	9.173±0.642^{c}	11.84±1.769^{c}

(g.m².day $^{-1}$)									
WPV (g·mm/m²·day·Pa)	2.29±0.444^{e}	7.48±0.329^{b}	10.30±0.195^{a}	2.32±0.155^{e}	2.45±0.189^{e}	6.64±0.196^{b}	5.68±0.860^{c}	2.50±0.176^{e}	3.74±0.560^{d}
Maximum Tensile Strength (MPa)	3.00±1.276^{ab}	3.14±0.229^{ab}	2.01±0.351^{bcd}	4.15±0.701^{a}	2.97±0.933^{ab}	0.67±0.163^{de}	0.53±0.043^{e}	1.01±0.676^{cde}	2.30±0.590^{bc}
Elongation (%)	132.20±35.057^{bc}	165.11±15.196^{bc}	204.50±28.861^{ab}	136.34±25.093^{bc}	130.90±11.457^{bc}	99.13±0.548^{c}	262.39±104.398^{a}	147.67±65.324^{bc}	137.62±34.31^{bc}
Flavonoids (mgQE.g^{-1})	0.05±0.003^{f}	12.03±0.152^{d}	17.43±0.153^{c}	24.30±0.871^{b}	39.16±0.072^{a}	0.07±0.004^{f}	0.07±0.001^{f}	1.19±0.066^{e}	1.51±0.005^{e}
Phenolic Compounds (mgGAE.g^{-1})	0.00^{g}	380.43^{d}	857.34^{c}	1084.10^{b}	1228.33^{a}	0.04^{e}	0.40^{e}	0.02^{f}	0.02^{f}
DPPH (%)	86.76±4.152^{a}	56.26±4.085^{d}	55.73±2.218^{d}	32.93±4.026^{e}	21.23±3.453^{f}	58.90±1.212^{cd}	58.20±5.729^{cd}	75.13±2.742^{b}	68.96±5.130^{bc}

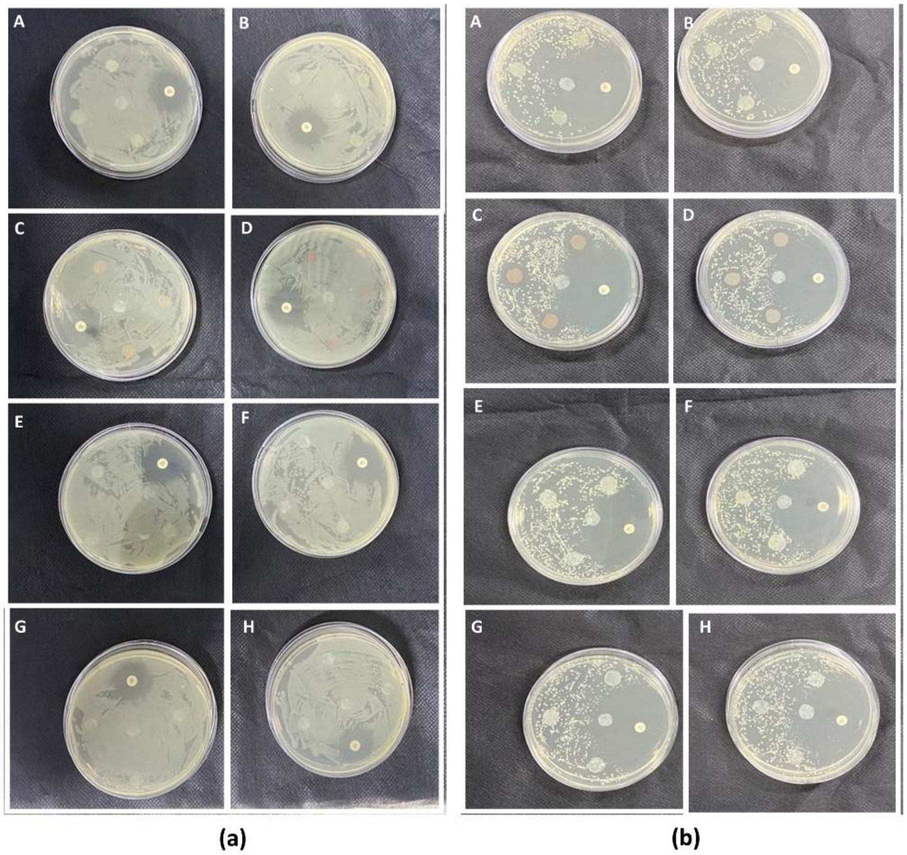

Figure S20. Analysis of the antimicrobial activity of the 8 active wound dressings developed (F2, F3. F4, F5, F6, F7, F8 and F9, referring to letters A, B, C, D, E, F, G and H, respectively): against (**a**) *E. coli* and (**b**) *S. aureus*.
